# *LACK OF SYMBIONT ACCOMMODATION* controls intracellular symbiont accommodation in root nodule and arbuscular mycorrhizal symbiosis in *Lotus japonicus*

**DOI:** 10.1371/journal.pgen.1007865

**Published:** 2019-01-03

**Authors:** Takuya Suzaki, Naoya Takeda, Hanna Nishida, Motomi Hoshino, Momoyo Ito, Fumika Misawa, Yoshihiro Handa, Kenji Miura, Masayoshi Kawaguchi

**Affiliations:** 1 Graduate School of Life and Environmental Sciences, University of Tsukuba, Tsukuba, Ibaraki, Japan; 2 College of Biological Sciences, University of Tsukuba, Tsukuba, Ibaraki, Japan; 3 Tsukuba Plant-Innovation Research Center, University of Tsukuba, Tsukuba, Ibaraki, Japan; 4 Graduate School of Science and Technology, Kwansei Gakuin University, Mita, Hyogo, Japan; 5 National Institute for Basic Biology, Okazaki, Aichi, Japan; 6 School of Life Science, Graduate University for Advanced Studies, Okazaki, Aichi, Japan; University of Munich, GERMANY

## Abstract

Nitrogen-fixing rhizobia and arbuscular mycorrhizal fungi (AMF) form symbioses with plant roots and these are established by precise regulation of symbiont accommodation within host plant cells. In model legumes such as *Lotus japonicus* and *Medicago truncatula*, rhizobia enter into roots through an intracellular invasion system that depends on the formation of a root-hair infection thread (IT). While IT-mediated intracellular rhizobia invasion is thought to be the most evolutionarily derived invasion system, some studies have indicated that a basal intercellular invasion system can replace it when some nodulation-related factors are genetically modified. In addition, intracellular rhizobia accommodation is suggested to have a similar mechanism as AMF accommodation. Nevertheless, our understanding of the underlying genetic mechanisms is incomplete. Here we identify a *L*. *japonicus* nodulation-deficient mutant, with a mutation in the *LACK OF SYMBIONT ACCOMMODATION* (*LAN*) gene, in which root-hair IT formation is strongly reduced, but intercellular rhizobial invasion eventually results in functional nodule formation. *LjLAN* encodes a protein that is homologous to Arabidopsis MEDIATOR 2/29/32 possibly acting as a subunit of a Mediator complex, a multiprotein complex required for gene transcription. We also show that LjLAN acts in parallel with a signaling pathway including LjCYCLOPS. In addition, the *lan* mutation drastically reduces the colonization levels of AMF. Taken together, our data provide a new factor that has a common role in symbiont accommodation process during root nodule and AM symbiosis.

## Author summary

Symbiosis between plants and beneficial microbes such as nitrogen-fixing bacteria and arbuscular mycorrhizal fungi has enabled plant colonization of new environments. Root nodule symbiosis with nitrogen-fixing rhizobia enables sessile plants to survive in a nitrogen-deficient environment. To establish the symbiosis, host plant cells need to accommodate rhizobia during nodule development, a process mediated by a plant-derived intracellular structure called the infection thread (IT). In this study, we show that *LACK OF SYMBIONT ACCOMMODATION* (*LAN*) is involved in intracellular rhizobia accommodation in the model leguminous plant *Lotus japonicus*. *LjLAN* encodes a putative subunit of Mediator complex, a multiprotein complex that has a fundamental role as an activator of gene transcription. Mutation analysis suggests that *LjLAN* is required for root hair IT formation, which enables swift and efficient rhizobial accommodation. Moreover, we show that *LjLAN* is required for symbiosis with arbuscular mycorrhizal fungi. These data add a new component to the molecular mechanism relevant to the establishment of root nodule and arbuscular mycorrhizal symbiosis.

## Introduction

Legumes can establish a symbiotic association with nitrogen-fixing bacteria through the formation of symbiotic root nodules. Nodulation is initiated by the rhizobia-derived lipo-chitooligosaccharidic nodulation (Nod) factors that trigger transient increases in calcium influx levels accompanied with calcium oscillation in the rhizobia-attached root hair cells, initiating dedifferentiation of the underlying cortical cells [1–3]. Studies using two model legumes, *Lotus japonicus* and *Medicago truncatula*, have revealed the basically conserved molecular mechanism that results in the progress of Nod factor signaling. In *L*. *japonicus*, Nod factor is recognized by two LysM receptor-like kinases NOD FACTOR RECEPTOR 1 (LjNFR1) and LjNFR5 [4–6], which induce a downstream signaling cascade. The Nod factor signaling pathway includes SYMBIOSIS RECEPTOR-LIKE KINASE (LjSYMRK), nucleoporins and cation channel proteins [7–11]. While loss-of-function mutations in components of the signaling pathway confer a complete nodulation deficiency phenotype, recent studies show that constitutive activation of either of *LjNFR1*, *LjNFR5* or *LjSYMRK* can induce spontaneous nodule formation in the absence of rhizobia [12, 13]. This indicates that at least these three kinases each possess a necessary and sufficient role for nodulation. Following the calcium oscillation, the *L*. *japonicus* CALCIUM CALMODULIN-DEPENDENT PROTEIN KINASE (LjCCaMK)/*M*. *truncatula* DOES NOT MAKE INFECTIONS 3 (MtDMI3) phosphorylates the transcription factor (TF) LjCYCLOPS/*M*. *truncatula* INTERACTING PROTEIN OF DMI3 (MtIPD3) [14–18]. Phosphorylated LjCYCLOPS then induces the *L*. *japonicus* RWP-RK type TF, NODULE INCEPTION (LjNIN), by directly binding to its promoter region [14, 19]. A number of nodulation-related genes now have been identified as direct targets of Lj/MtNIN, including genes encoding the NUCLEAR FACTOR (NF)-Y subunits [20]. Root cortical proliferation is induced by constitutive expression of either of phosphorylated LjCYCLOPS, LjNIN or LjNF-Y subunits in the absence of rhizobia [14, 20, 21], indicating that induction of the LjCYCLOPS>LjNIN>LjNF-Y hierarchical transcription cascade is sufficient to initiate nodulation. Several data indicate that cytokinin signaling is another essential regulator of nodulation, and that Lj/MtNIN is a downstream component of the cytokinin signaling pathway, as indicated by findings that functional cytokinin receptor is required for rhizobia- and cytokinin-dependent *Lj/MtNIN* induction [22, 23]. It was recently shown that MtNIN directly binds to the promoter region of the *CYTOKININ RESPONSE 1* (*MtCRE1*) gene encoding a cytokinin receptor and promotes its expression at root cortex [21, 24]. This result indicates that there is a positive feedback loop between MtNIN and cytokinin signaling. In addition to its activating role in nodulation, in some contexts LjNIN can negatively regulate nodule organogenesis through direct activation of *CLE-ROOT SIGNAL 1* (*LjCLE-RS1*) and *-RS2* that function as putative root-derived signals in long-distance inhibitory signaling of nodulation [23].

Accommodation of rhizobia within host cells is indispensable for the establishment of root nodule symbiosis; therefore, proliferating cortical cells need to be invaded by rhizobia at the appropriate time during nodulation. In *L*. *japonicus* and *M*. *truncatula*, the rhizobial invasion process starts from the tip of the root hair associated with root hair curing. Rhizobia invade proliferating cortical cells through a plant-derived intracellular tube-like structure called the infection thread (IT), and are finally released into host cells by endocytosis [25–27]. The signaling cascade initiating nodule organogenesis is also essential for the rhizobial invasion process, because in most cases root-hair IT formation is severely retarded if key proteins in the signaling pathway are mutated. A recent study demonstrated that, in addition to Nod factor, rhizobia-derived exopolysaccharides have a crucial role in the rhizobial accommodation process via interactions with the EXOPOLYSACCHARIDE RECEPTOR 3 (LjEPR3), a LysM receptor-like kinase that is paralogous to LjNFR1 [28]. The Nod factor signaling seems to have a role to induce the *LjEPR3* expression at the epidermis. Overall, one signaling pathway achieves two qualitatively and spatially different phenomena, that is, rhizobial root hair accommodation at the epidermis and nodule organogenesis at the cortex. Studies using an epidermal-specific expression system indicated that this can be explained by a difference in the tissue-specific requirements of the genes involved in Nod factor signaling [29, 30]. In addition, cell-to-cell communication between the epidermis and cortex may be involved [31]. In terms of transcriptional regulation, a direct target of LjNIN, *NODULATION PECTATE LYASE* (*LjNPL*) has been implicated in the degradation of plant cell walls, and is required for normal root-hair IT formation [32]. Thus, LjNIN may participate in rhizobia accommodation through activation of genes relevant to root-hair IT formation, such as *LjNPL*. MtNF-Y subunits seem to be involved in rhizobial accommodation processes as well as nodule organogenesis. LjNIN can also directly induce *LjEPR3* expression; the LjNIN>*LjEPR3* cascade appears to control rhizobia infection process [33]. In particular, *ETHYLENE RESPONSIVE FACTOR REQUIRED FOR NODULATION 1* (*MtERN1*) that encodes a TF involved in root-hair IT formation together with its close homologue MtERN2, was shown to be a direct target of MtNF-Y subunits [34–36]. Moreover, LjCYCLOPS has a role directly inducing *LjERN1* expression [37]. In addition, recent studies show that epidermal cytokinin signaling appears to have a negative role in root hair IT formation [38–40]. Despite these advances in our understanding of the molecular mechanism of nodule organogenesis and the rhizobia accommodation process, our understanding of the mechanism remains incomplete, indicating that further components await discovery.

Symbiosis between plants and arbuscular mycorrhizal fungi (AMF) is another widely observed plant-microbe mutual relationship known as AM symbiosis. The plant regulatory pathway for AM symbiosis has been shown to share some components, called common symbiosis pathway (CSP) genes, of its genetic pathway with root nodule symbiosis [3, 41]. Based on current data, the role of CSP genes is thought to mostly relate to generate calcium signaling and make a read-out, which occurs commonly during the two symbioses. Both symbioses are strongly impaired by a mutation in the CSP genes such as *LjSYMRK*, *LjCCaMK* and *LjCYCLOPS*. In AM symbiosis the LjCCaMK-LjCYCLOPS module responds to calcium oscillation, transmitting a signal to the downstream pathway, that results in the formation of symbiotic organs such as the arbuscule. LjCYCLOPS/MtIPD3 physically interacts with Lj/MtDELLA to form the LjCCaMK/MtDMI3-LjCYCLOPS/MtIPD3-Lj/MtDELLA complex that directly induces the *REDUCED ARBUSCULAR MYCORRHIZA 1* (*Lj/MtRAM1*) GRAS-type TF during AM symbiosis, which is required for arbuscule branching [42–44].

In the present study, we identify a *L*. *japonicus* mutant with a mutation in the *LACK OF SYMBIONT ACCOMMODATION* (*LjLAN*) gene. Observations of rhizobia infection/invasion patterns together with nodulation foci show that in *lan* mutant a developmental program of nodulation proceeds in the absence of root-hair IT formation, where rhizobia enter into roots through an intercellular invasion system. The *LjLAN* gene encodes a protein that is putatively orthologous to Arabidopsis MEDIATOR 2/29/32 (AtMED2/29/32) constituting a Mediator complex. Moreover, the *lan* mutation reduces symbiosis with AMF. These data suggests LjLAN acts as a putative transcriptional regulatory module required for the establishment of both root nodule and AM symbiosis.

## Results

### Isolation of a *L*. *japonicus* mutant with a defect in nodulation

**Fig 1:**
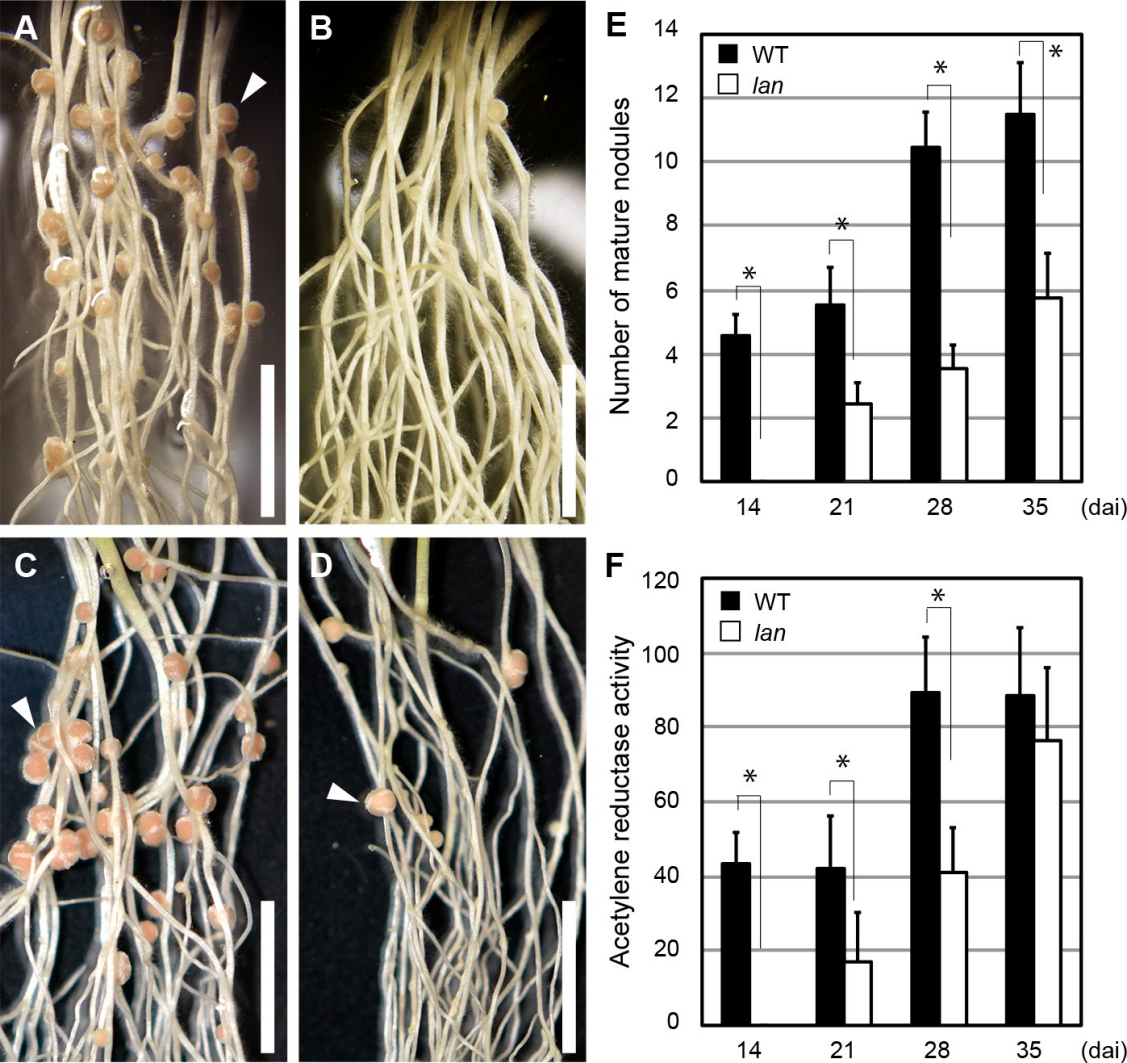
The effect of the *lan* mutation on nodulation. (A-D) Nodulation phenotype of WT MG-20 (A and C) and of the *lan* (B and D) roots. Roots were observed at 14 (A and B) and at 21 (C and D) dai. (E) The numbers of mature nodules formed in WT MG-20 and in the *lan* plants from 14 to 35 dai. Mature nodules were judged by several indicators including sizes, colors and lenticels formation (n = 12–14 plants). (F) Acetylene reductase activity (ARA) (nmol/ h per plant) of WT MG-20 and the *lan* plants from 14 to 35 dai. ARA was measured using the same nodules judged as mature ones in E. Arrowheads indicate mature nodules. Scale bars: 5 mm. Error bars indicate SD. *P < 0.05 by Student’s *t* test.

To better understand the molecular mechanisms associated with the control of nodulation, we undertook a screen for nodulation-deficient mutants from EMS-treated *L*. *japonicus* wild-type (WT) MG-20 plants. From this screen we isolated a mutant with a mutation in the gene that we named *lack of symbiont accommodation* (*lan*) based on the nodulation-deficient phenotype. F1 plants derived from a cross between *lan* and the WT MG-20 parental line showed normal nodulation. In the F2 population, normal-nodulation and nodulation-deficient plants segregated in an approximately 3:1 ratio (58 normal-nodulation and 18 nodulation-deficient plants). Thus, the *lan* mutation is inherited as a recessive trait. In *L*. *japonicus*, mature nodules can be characterized by several morphological and physiological indicators, including nodule size, color, lenticel formation, and nitrogen fixation activity. In WT plants, formation of mature nodules was recognizable at the latest 14 days after inoculation of *Mesorhizobium loti* (dai) (Fig 1A, 1C, 1E and 1F). In contrast, in the *lan* mutant, no mature nodules were formed at the corresponding stage (Fig 1B and 1E). Formation of mature nodules could be observed at 21 dai, and their number gradually increased over time (Fig 1D and 1E), although the number was consistently lower than WT. Analysis of acetylene reductase activity per plant showed that nodules formed on the mutant roots at a later stage, such as 35 dai, were comparable to those of WT (Fig 1F). Therefore, in terms of nitrogen fixation activity, the mutant nodules formed at the stage appeared to be functional.

### Nodulation is not associated with infection thread formation in the *lan* mutant

**Fig 2:**
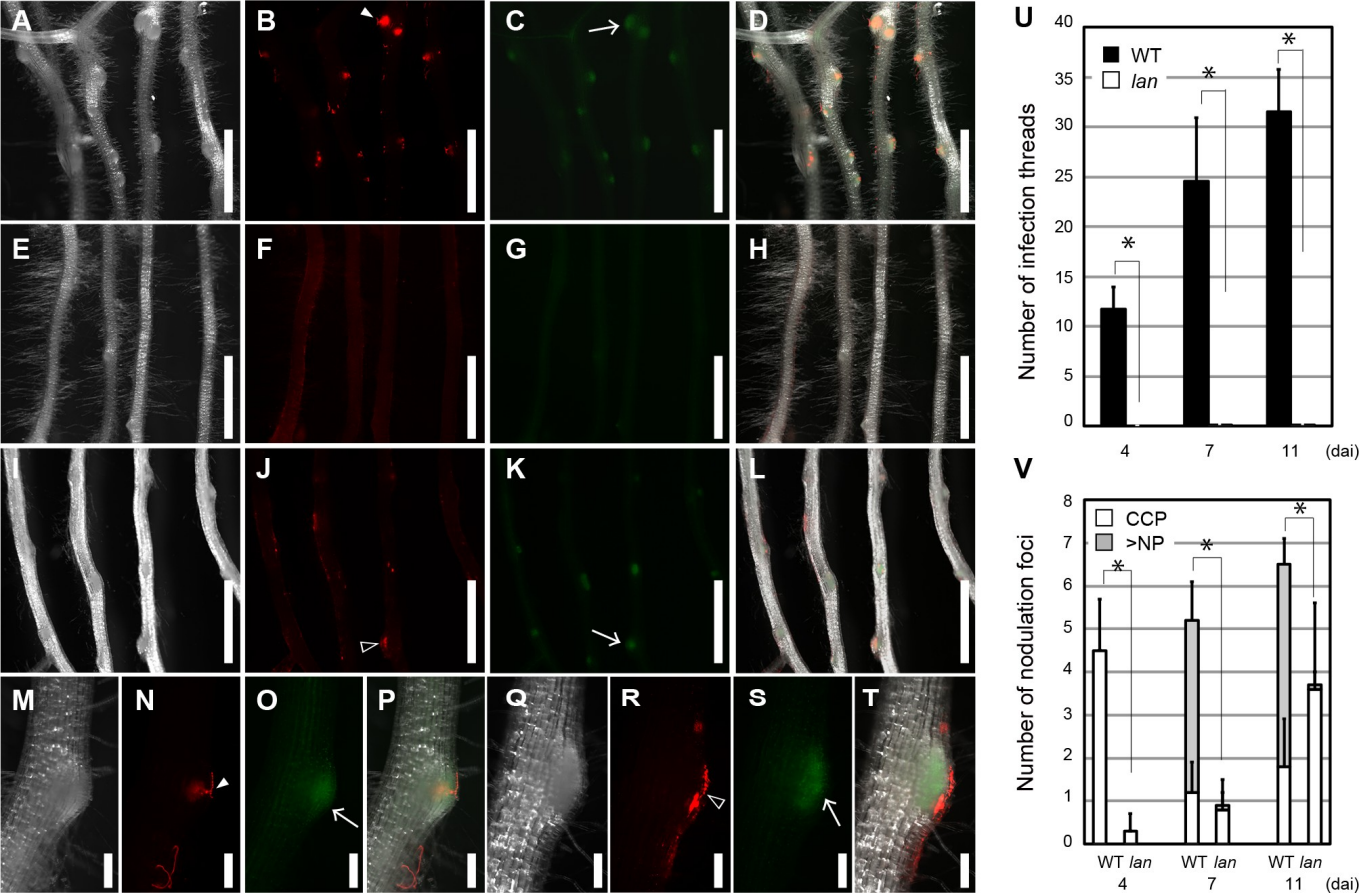
The effect of the *lan* mutation on root-hair ITs formation and on early nodule development. (A-T) Root-hair ITs formation and auxin response patterns of the *DR5*:*GFP-NLS*/WT MG-20 plants (A-D and M-P) and of the *DR5*:*GFP-NLS*/*lan* plants (E-L and Q-T). Roots were observed at 4 (A-H and M-P) and at 11 (I-L and Q-T) dai. *M*. *loti* MAFF303099 constitutively expressing *DsRED* was used for inoculation. (A, E, I, M and Q) Bright-field images of roots. (B, F, J, N and R) Red fluorescence images of the same roots shown in A, E, I, M and Q, respectively. (C, G, K, O and S) Green fluorescence images of the same roots shown in A, E, I, M and Q, respectively. (D, H, L, P and T) Merged images of roots. Closed and open arrowheads respectively indicate root-hair ITs and accumulation of rhizobia. Arrows indicate auxin responses. (U) The numbers of root-hair ITs formed in the *DR5*:*GFP-NLS*/WT MG-20 and in the *DR5*:*GFP-NLS*/*lan* plants (n = 10 plants). Root-hair ITs were identified by DsRED signals derived from the transgenic rhizobia. (V) The numbers of sites of cortical cell proliferation (CCP) and of nodule primordia (NP) (n = 10 plants). CCP was identified by *GFP-NLS* signals that were expressed under the control of *DR5*. When cortical cells appeared bulged by the progress of several rounds of cell division, the sites were judged as NP. Scale bars: 1 mm (A-L); 100 μm (M-T). Error bars indicate SD. Student’s t-test was performed by comparing total data points, namely, CCP+NP. *P < 0.05.

In order to characterize the effect of the *lan* mutation on root-hair IT formation and early nodulation, we used two fluorescent-based markers to visualize infection and nodulation foci. A *M*. *loti* strain expressing *DsRED* was used to mark root-hair ITs. During nodule development, a preferential auxin response is observed in proliferating cortical cells and bulge of nodule primordia [45–47]. Thus, we tried to quantify the sites of nodulation foci (cortical cells proliferation and nodule primordia) based on the expression of a reporter gene under the control of auxin responsive element DR5. To visualize the nodulation foci in *lan* mutant, we produced *DR5*:*GFP-NLS/lan* plants by crossing *DR5*:*GFP-NLS*/WT transgenic plants [45] with the *lan* plants. In *DR5*:*GFP-NLS*/WT plants, the formation of root-hair ITs was recognizable at 4 dai, and cortical cells located under some of the ITs started to proliferate (Fig 2A–2D, 2M–2P, 2U and 2V). In contrast, root-hair ITs were barely observed in the *DR5*:*GFP-NLS/lan* plants during the corresponding time scale (Fig 2E–2H and 2U). In the *DR5*:*GFP-NLS/lan* plants, although root-hair ITs were almost undetectable at all time points tested, we found some sites of auxin response, which implied cortical cell proliferation and the formation of nodulation foci (Fig 2I–2L, 2Q–2T, 2U and 2V). The number of nodulation foci gradually increased over time after inoculation (Fig 2V). In most cases, the occurrence of nodulation foci was accompanied with bright DsRED signals suggesting the accumulation of rhizobia at the surface of developing nodules. These results indicate that in the *lan* mutant the nodulation developmental program can be initiated in the absence of root-hair IT formation. Some mutants impaired in root-hair ITs formation tend to develop an excess number of small uninfected nodule primordia [48–50]. Even in the later nodulation stage such as 45 dai, the formation of such small uninfected nodule primordia were not observed in the *DR5*:*GFP-NLS/lan* plants (S1A Fig). In addition, inoculation of *M*. *loti nodC* mutants, which could not synthesize functional Nod factors, did not result in making any nodules in the *lan* mutant as well as WT (S1B Fig). Thus, the nodulation in the *lan* mutant depends on Nod factor signaling.

During nodulation, a series of calcium oscillations, defined as calcium spiking, in responsive cells is induced in response to the rhizobia-derived Nod factor [51, 52]. A normal calcium spiking pattern could be observed in the *lan* root hair cells following application of purified Nod factor (S2 Fig), indicating that in the *lan* mutant, nodulation signaling upstream of the calcium spiking response is unaffected.

### Rhizobia enter into roots through the intercellular invasion system in the *lan* mutant

**Fig 3:**
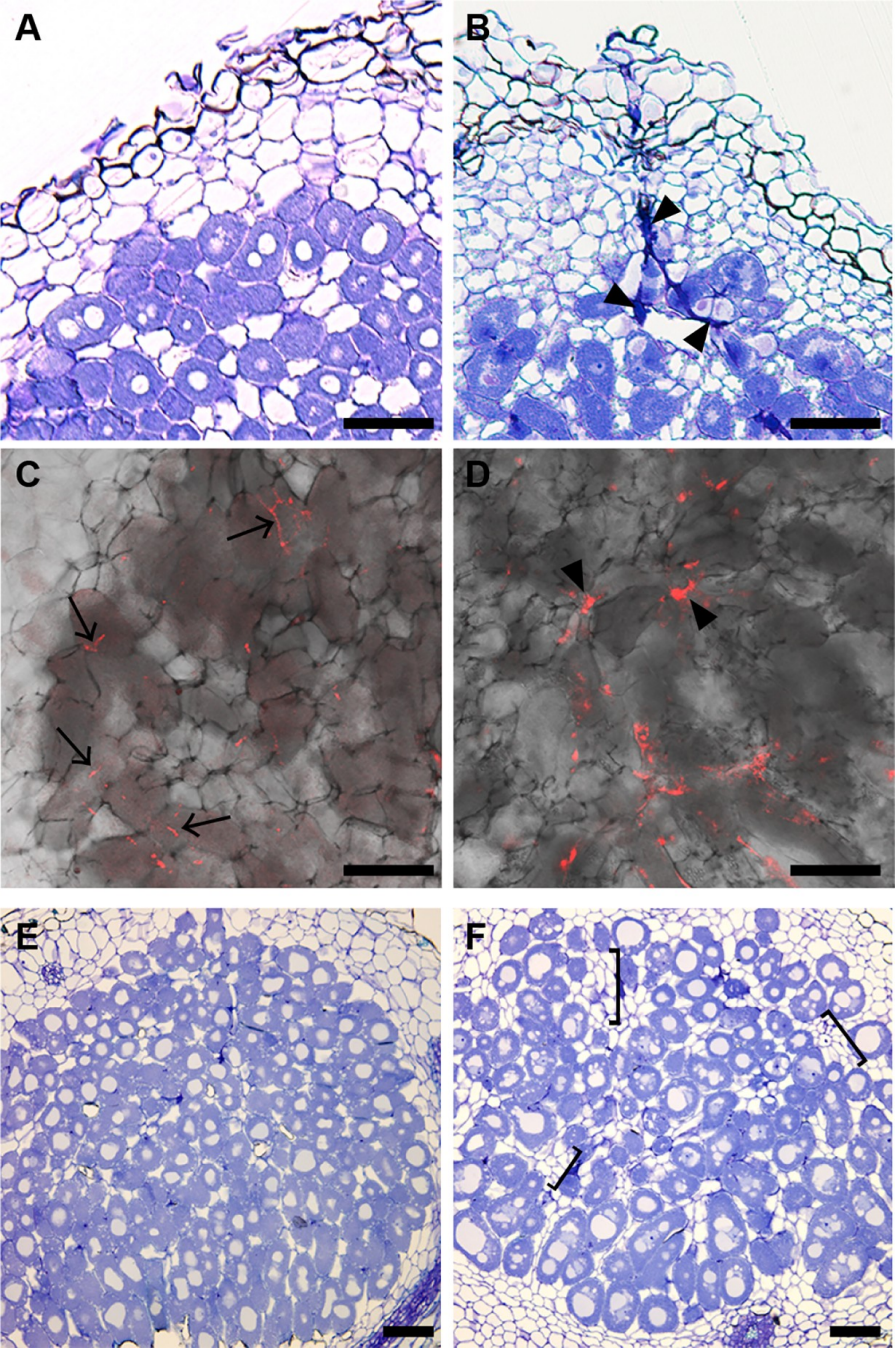
The effect of the *lan* mutation on pattern of rhizobial invasion to nodules. (A and B) Sections through nodules of WT MG-20 (A) and of *lan* (B) at 21 dai, which were stained with 0.05% Toluidine blue. (C and D) Confocal images of sections through nodules of WT MG-20 (C) and of *lan* (D) at 33 dai. *M*. *loti* MAFF303099 constitutively expressing *DsRED* was used for inoculation. (E and F) Sections through nodules of WT MG-20 (E) and of *lan* (F) at 35 dai, which were stained with 0.05% Toluidine blue. Sections with maximum diameter are shown. At least 5 independent WT and *lan* nodules were analyzed to conclude their character. Arrowheads indicate intercellular bacteria pocket. Arrows indicate cortical-ITs. Brackets indicate clusters of uninfected cells. Scale bars: 50 μm.

In *L*. *japonicus DR5*:*GFP-NLS*/WT plants, rhizobia use the root-hair IT-mediated intracellular invasion system to enter into roots (S3A and S3C Fig) [53]. In *DR5*:*GFP-NLS/lan* plants, despite strongly impaired root-hair ITs formation (Fig 2U), cortical cell proliferation is induced, which results in the formation of nitrogen-fixing nodules (Figs 1E, 1F and 2V), raising the question of how rhizobia enter into roots in the *lan* mutant. The accumulation of rhizobia on the epidermis of nodule primordia suggested that rhizobia might enter developing nodules through intercellular invasion system as was previously reported in other *L*. *japonicus* mutants (Fig 2Q–2T and S3B and S3D Fig) [11, 54]. Thus, in order to clarify rhizobial localization in nodules, we examined sections of nodules. In the mutant nodules, a dense population of rhizobia was observed in some intercellular spaces (Fig 3A–3D). This bacteria localization pattern is reminiscent of that defined as pocket of intercellular bacteria seen in the several *L*. *japonicus* mutants, where rhizobia enter nodules predominantly through intercellular invasion system [11, 50, 54–56]. In WT nodules rhizobia enter nodule cells through cortical-ITs (Fig 3C) [56]. On the other hand, we could not determine the presence of cortical-ITs in the mutant nodules. An observation of *lan* mutant nodule sections of relatively later stage showed that the number of rhizobia-colonized cells were evidently reduced compared with WT (Fig 3E and 3F). In WT nodules, rhizobia-colonized cells were tightly packed at the inner region of nodules (Fig 3E). On the other hands, in the *lan* mutant, clusters of uninfected cells were located between rhizobia-colonized cells (Fig 3F). Thus, the *lan* mutation can affect rhizobia accommodation process throughout nodule development.

### *LjLAN* encodes a putative subunit of the Mediator complex

**Fig 4:**
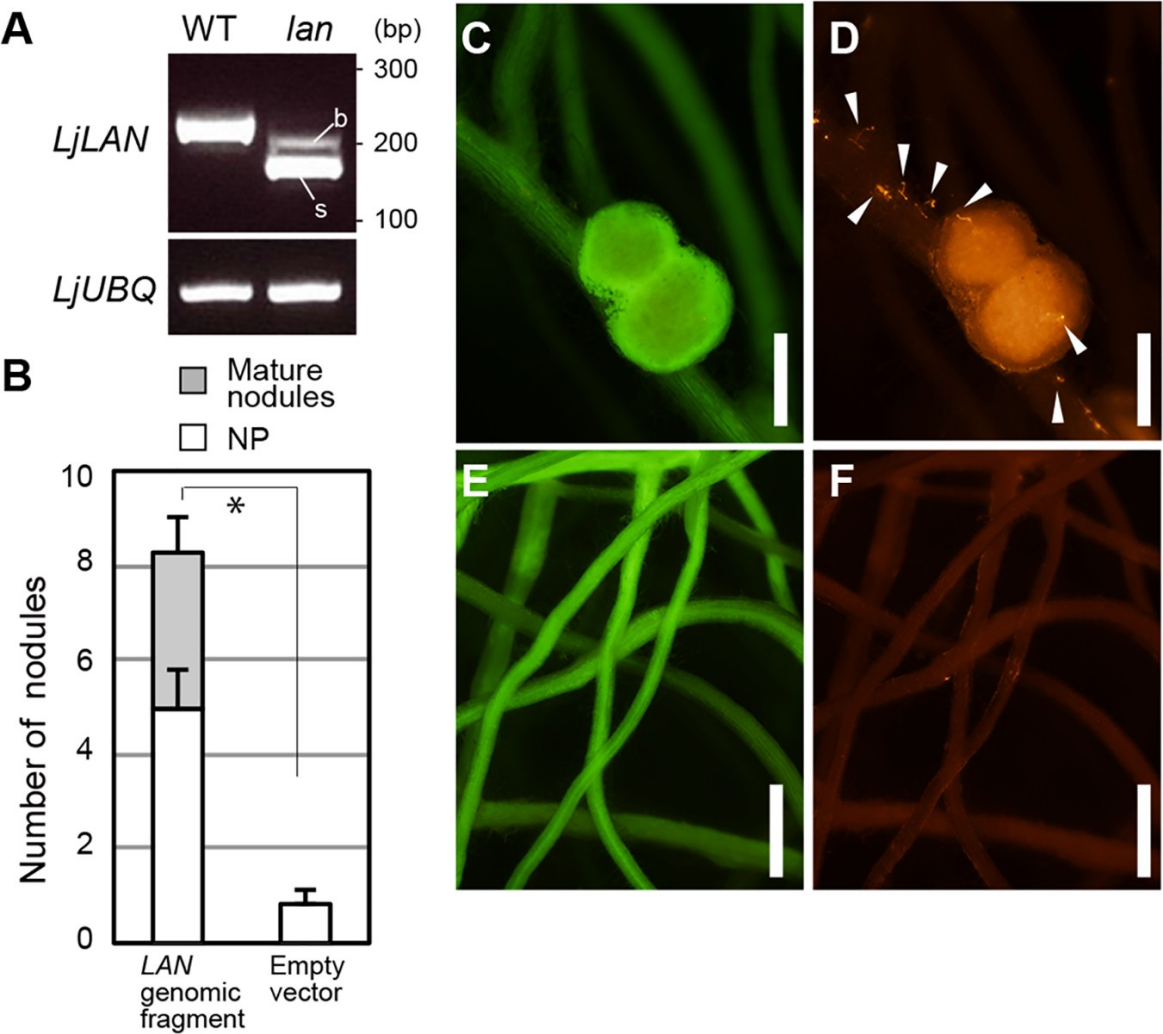
*LjLAN* expression pattern and complementation analysis. (A) RT-PCR analysis of the *LjLAN* gene. *LjUBQ* was used as the RNA loading control. The locations of primer sets used for PCR is shown in S5 Fig. cDNA was prepared from total RNAs from 7 dai roots. In *lan*, two transcripts, a minor transcript with big size (b) and a major transcript with small size (s), are detected, whose sequences are shown in S5B–S5F Fig. Complementation of the *lan* nodulation phenotype. (B) Average nodule number in the *lan* mutant with transgenic roots containing respective constructs at 14 dai (n = 13–18 plants). NP, nodule primrdia. (C-F) Representative transgenic hairy roots of *lan* carrying a 5.8-kb genomic fragment encompassing the entire *LjLAN* locus (C and D) and a control empty vector (E and F). (C and E) Green fluorescence images of roots. (D and F) Red fluorescence images of the same roots shown in C and E. *M*. *loti* MAFF303099 constitutively expressing *DsRED* was used for inoculation. Transgenic roots were identified by the expression of GFP-LjLTI6b. Arrowheads indicate root-hair ITs. Scale bars: 1 mm. Error bars indicate SD. Student’s t-test was performed by comparing total nodule number. *P < 0.05 by Student’s *t* test.

To understand the molecular function of *LjLAN*, we first sought to isolate the gene by a positional cloning approach. This mapped the *LjLAN* locus to a region between the simple sequence repeat (SSR) markers TM0216 and TM0135 on chromosome 3 (S4 Fig). Subsequent genome-resequencing of the *lan* mutant identified an A-to-T nucleotide substitution that occurs in the acceptor site of an intron located upstream of the gene, chr3.CM0112.280.r2.d (S5 Fig). In the mutant, the nucleotide substitution causes the production of two transcripts smaller than that of WT (Figs 4A and S5). We sequenced each mutant transcript, and found that in both cases intron mis-splicing spliced out a DNA region encompassing the original initiation codon of the gene. In addition, in the *lan* mutant no coding sequence was predictable in the locus. Thus, it is reasonable to suppose that the *lan* mutation causes a complete loss of function of the gene. The mutant two transcripts were detectable all time points tested after inoculation (S6A Fig). The *lan* mutation reduced the expression of the gene (S6B Fig). To verify if this gene is responsible for the *lan* mutation, a 5.8-kb genomic fragment containing the WT gene was introduced into the mutant by *Agrobacterium rhizogenes*-mediated hairy root transformation. The introduction of the fragment into the mutant rescued the phenotype, resulting in the formation of normal number of nodules at 14 dai (Fig 4B–4F), and normal root-hair ITs formation (Fig 4C–4F). The *LjLAN* gene encodes an uncharacterized protein of 145 amino acids that is putatively orthologous to AtMED2/29/32, a putative subunit of the Mediator complex (S7 Fig). It is generally thought that the Mediator complex, which consists of a large number of subunits, plays a role as a bridge between promoter-bound TFs and RNA polymerase II to activate gene transcription [57–59]. Indeed AtMED2 was shown to be required for the recruitment of RNA polymerase II [60]. *AtMED2* could rescue the *lan* mutation when it was constitutively expressed by *LjUBQ* promoter (S8 Fig), suggesting that the LjLAN has a function similar to AtMED2. The phylogenetic analysis identified a homologue of LjLAN in *L*. *japonicus*, which was designated as LjLAN LIKE (S7 Fig); the similarity and identity values are respectively 94.6% and 81.7%.

The expression pattern of the *LjLAN* and *LjLAN LIKE* gene remained constant in some vegetative and reproductive organs investigated (S9A and S9B Fig). To gain insights into the role of the *LjLAN* gene during nodulation, we examined the time course expression pattern after inoculation of *M*. *loti*. *LjLAN* expression was largely constant during nodulation when whole roots were assayed by RT-qPCR (S9C Fig). However, an approximately 2-fold induction of *LjLAN* expression was detected in root segments where proliferating cortical cells were enriched (S9C Fig). Furthermore, reporter gene analysis using *ProLjLAN*:*GUS plus* construct showed that during nodulation the GUS activity was detectable at epidermis with curled root hairs, proliferating cortical cells and nodule primordia (S10 Fig). The GUS activity was also observed at lateral roots.

The *lan* mutant used for above-mentioned analyses has Miyakojima MG-20 genetic background. We obtained a plant with Gifu B-129 genetic background in which a retrotransposon, *LOTUS RETROTRANSPOSON 1* (*LORE1*) [61, 62], was inserted in the middle region of coding sequence of *LjLAN* gene, causing an occurrence of premature stop codon in the mutant (S11A and S11B Fig). Consequently, we found that the plants have the truncated protein of LjLAN lacking C-terminal part of it (S11B Fig). Unexpectedly, the *LORE1*-tagged mutant showed normal nodulation phenotypes (S11C and S11D Fig). In order to interpret the observation, we raised two possibilities. First, the effects of *lan* mutation was observable in an ecotype-specific manner. The second possibility was that the truncated LjLAN that was produced in the *LORE1*-tagged mutant was functional. To verify them, we introduced modified LjLAN (LjLANΔC), in which amino acid residues constituting C-terminal part of LjLAN were deleted (S11B Fig), into *lan* mutant. LjLANΔC could rescue the *lan* mutation to the extent of same level of the introduction of control intact LjLAN (S8 Fig). We then created stable transgenic plants with nucleotide deletions or insertions in the middle region of coding sequence of *LjLAN* gene by CRISPR-Cas9 genome-editing system. In the transgenic plants, the frame-shifted mutations caused the deletion of amino acid residues constituting C-terminal part of LjLAN (S11B and S11E Fig). The nodulation phenotypes of the transgenic plants were indistinguishable from WT plants (S11F and S11G Fig). These results indicate that C-terminal part of LjLAN is not essential for the LjLAN function. Therefore, the lack of phenotype of the *LORE1*-tagged mutant can be explained by the retention of LjLAN function rather than an ecotype difference. The *LORE1*-tagged mutation did not affect the expression of *LjLAN* (S6B Fig).

### LjLAN acts in parallel with a signaling pathway including LjCYCLOPS

**Fig 5:**
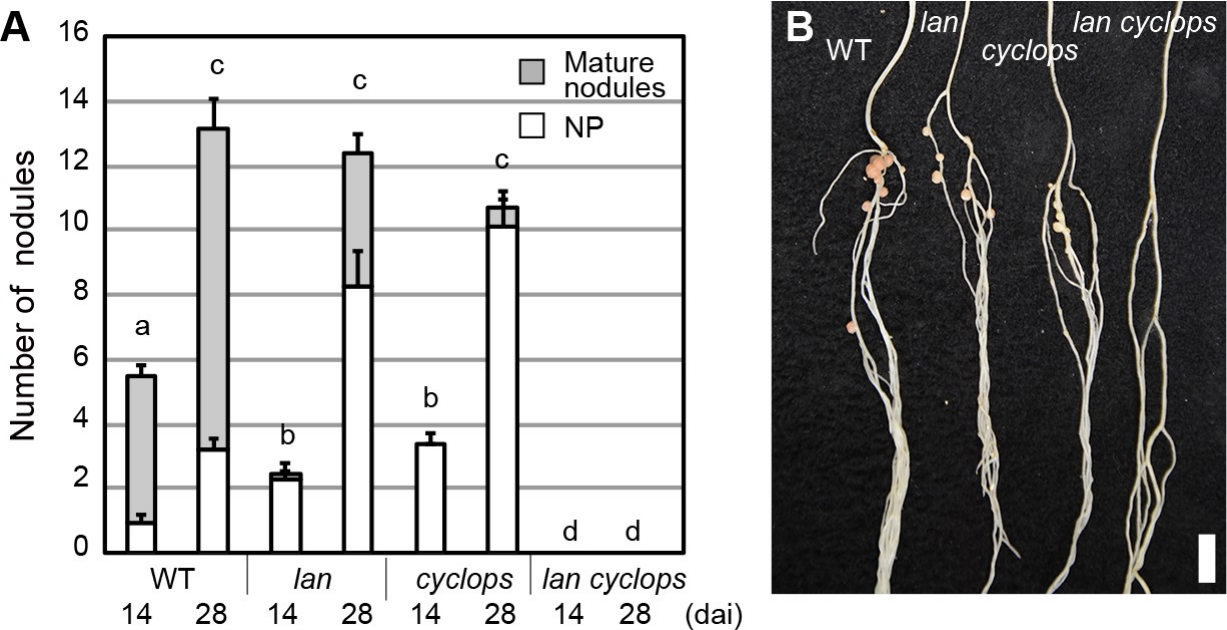
Nodulation phenotype of *lan cyclops* double mutant. (A) Average nodule number in the WT MG-20, *lan*, *cyclops-6*, *lan cyclops-6* plants at 14 and 28 dai (n = 12 plants). NP, nodule primrdia. (B) Nodulation phenotype of WT MG-20, *lan*, *cyclops-6*, *lan cyclops-6* plants at 28 dai. Scale bar: 5 mm. Tukey’s test was performed by comparing total nodule number. Columns with the same lower-case letter indicate no significant difference. P < 0.05.

After decoding calcium spiking followed by rhizobial infection, LjCYCLOPS has an important role in root nodule symbiosis, as it regulates both rhizobial infection and nodule organogenesis through induction of different downstream target genes [14, 37]. *cyclops* mutants retain nodulation to some extent [63], providing an accessible baseline for screen for second mutations influencing the *cyclops* nodulation defects. We then created *lan cyclops* double mutant. Of note, the *lan cyclops* double mutant plants showed a complete non-nodulating phenotype, different from each single mutant (Fig 5).

**Fig 6:**
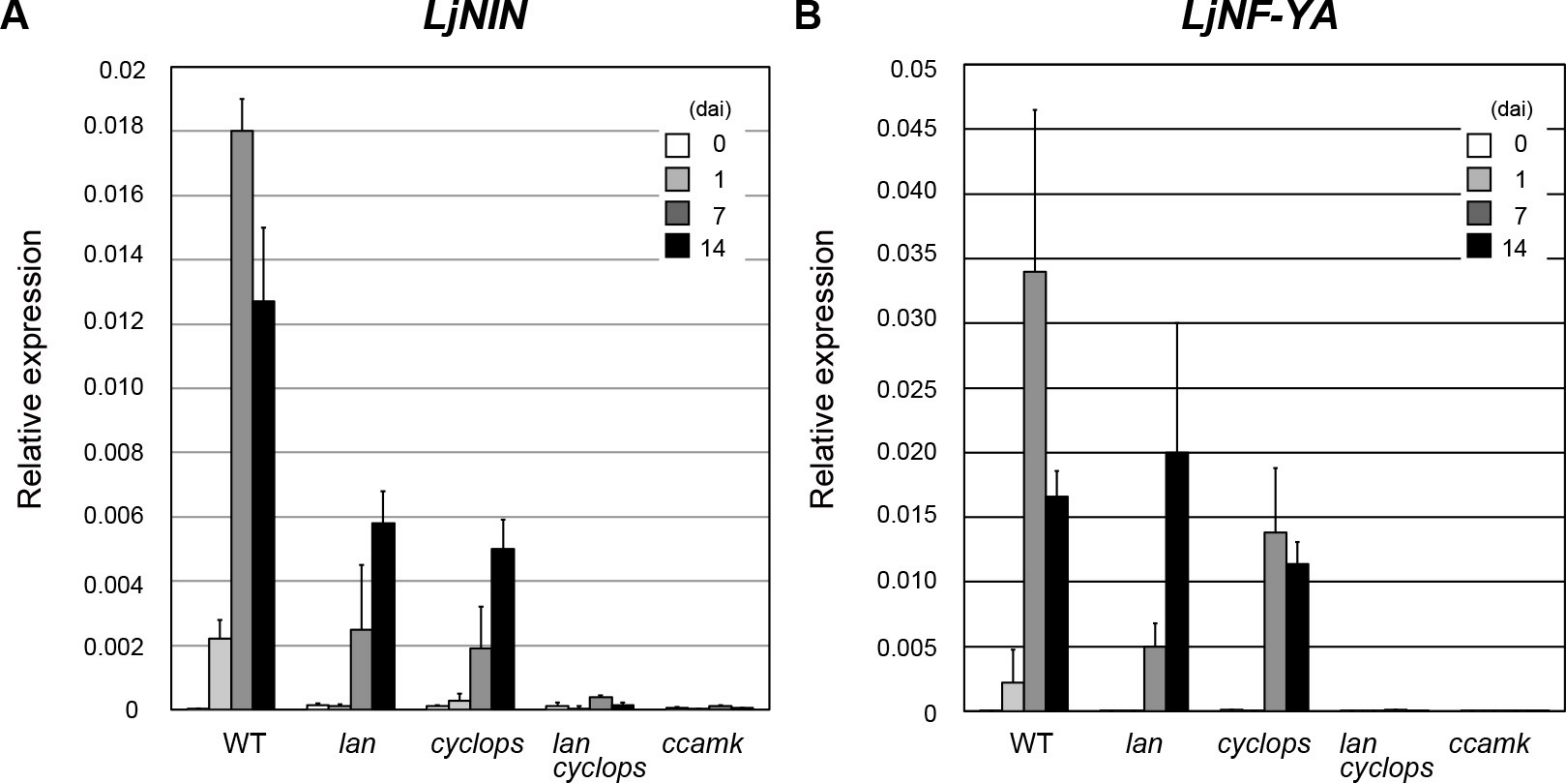
The effect of the *lan* and *cyclops* mutation on the expression of nodulation marker genes. (A and B) Real-time RT-PCR analysis of *LjNIN* (A) and *LjNF-YA* (B) expression in uninoculated WT MG-20, *lan*, *cyclops-6*, *lan cyclops-6* and *ccamk-14* roots (0), and in inoculated roots at 1, 7 and 14 dai. cDNA was prepared from total RNAs from whole roots. *LjUBQ* was used to assess the relative expression of each gene. Error bars indicate SD.

To gain insight into the potential relationship between *LjLAN* and *LjCYCLOPS* with respect to gene expression, we investigated the two nodulation-related genes expression, *LjNIN* and *LjNF-YA*. *LjNIN*, a direct target of LjCYCLOPS, has a pivotal role in the transcriptional cascade that is required for both nodule formation and rhizobial infection [19, 20], and *LjNF-YA* has been shown to be a direct target of LjNIN [20]. Confirming previous reports, we found that expression of *LjNIN* and *LjNF-YA* was strongly induced throughout nodulation stages investigated (Fig 6A and 6B) [19, 23, 31]. We found that in the *lan* and *cyclops* mutants the induction level of *LjNIN* was consistently weaker than that in WT along the time course after inoculation (Fig 6A). However, although the *lan* and *cyclops* mutation suppressed *LjNF-YA* induction at 1 and 7 dai, the induction level in *lan* and *cyclops* roots at 14 dai was largely comparable to that in WT roots of the corresponding stage (Fig 6B). Furthermore, in the *lan cyclops* double mutant, the expression of the two genes were strongly impaired at all time point tested as well as *ccamk* mutant. Together with *lan cyclops* nodulation phenotype, these results indicate that LjLAN acts in parallel with LjCYCLOPS for the control of key nodulation-related genes expression.

### The *lan* mutation affects symbiosis with arbuscular mycorrhizal fungi

**Fig 7:**
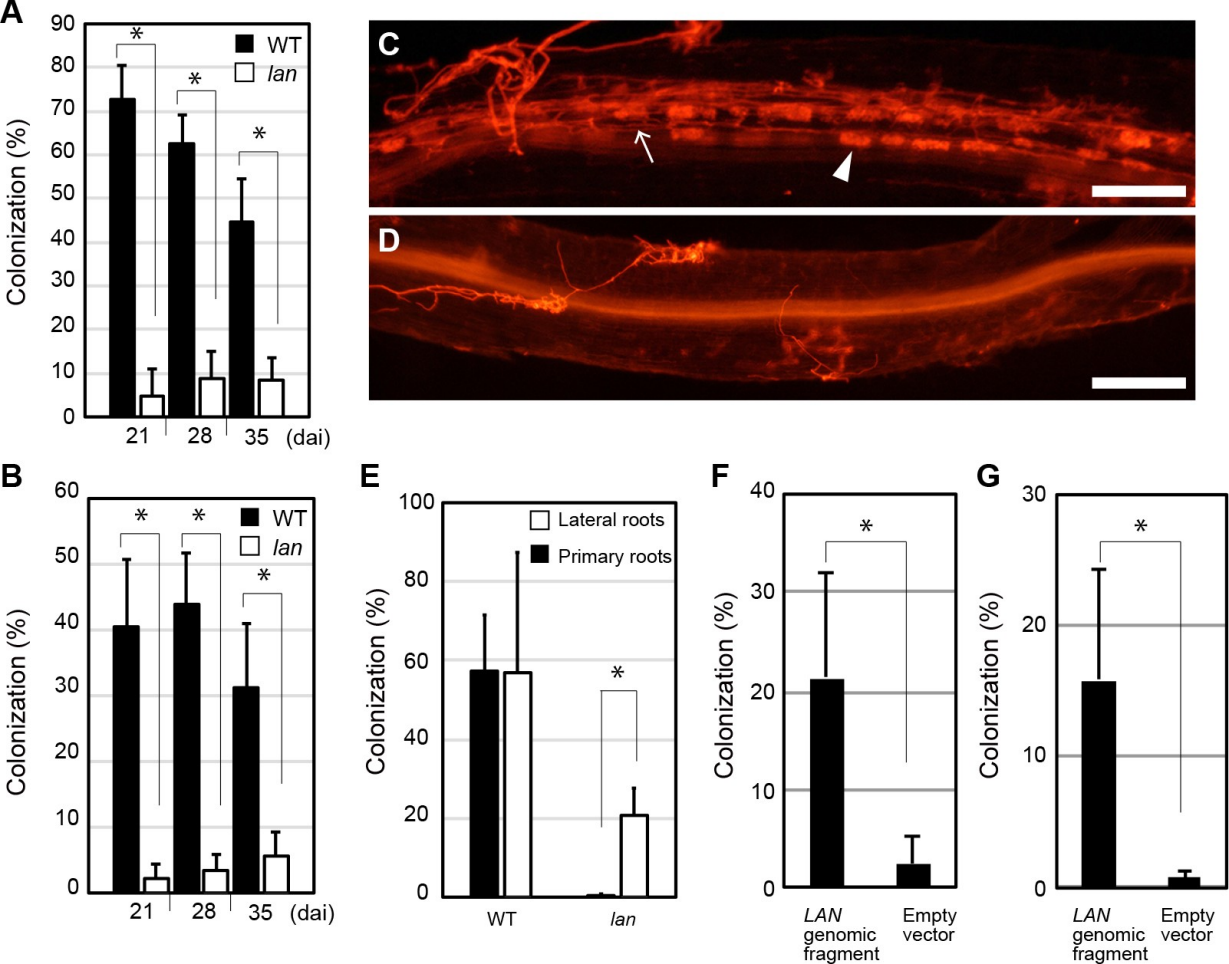
The effect of the *lan* mutation on symbiosis with AMF. (A and B) *R*. *irregularis* colonization ratio of hyphae (A) and arbuscules (B) from 21 to 35 dai. (n = 6 plants). (C and D) Hyphae and arbuscules formation of WT MG-20 (C) and *lan* (D) whole roots at 21 dai. AMF structures were stained with wheat germ agglutinin (WGA)-Alexa Fluor 594 (Alexa594) to detect *R*. *irregularis*. (E) *R*. *irregularis* colonization ratio of hyphae in primary and lateral roots at 21 dai (n = 8 plants). (F and G) Average colonization ratio of hyphae (F) and arbuscules (G) in the *lan* mutant with transgenic roots having primary and lateral roots, containing respective constructs at 21 dai (n = 6 plants). Representative transgenic hairy roots of *lan* carrying a 5.8-kb genomic fragment encompassing the entire *LjLAN* locus or a control empty vector were analyzed. Transgenic roots were identified by the expression of GFP-LjLTI6b. Arrowhead and arrow respectively indicate arbuscule and hypha. Scale bars: 200 μm. Error bars indicate SD. *P < 0.05 by Student’s *t* test.

**Fig 8:**
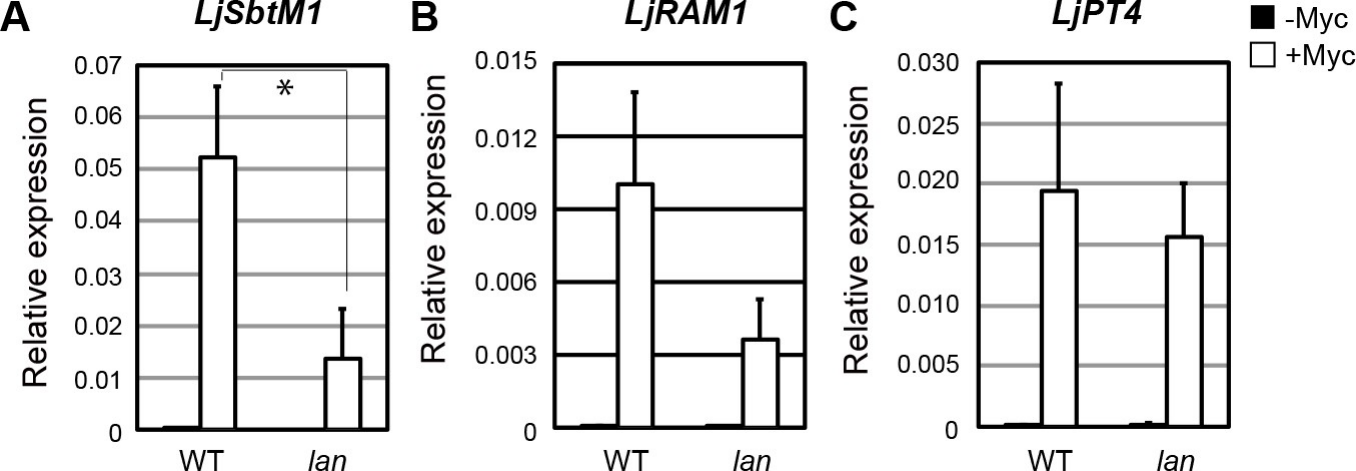
The effect of the *lan* mutation on the expression of the AM symbiosis marker genes. (A-C) Real-time RT-PCR analysis of *LjSbtM1* (A), *LjRAM1* (B) and *LjPT4* (C) expression in uninoculated (-Myc) and in 21 dai inoculated (+Myc) roots. cDNA was prepared from total RNAs from whole roots. *LjUBQ* was used to assess the relative expression of each gene. Error bars indicate SD. *P < 0.05 by Student’s *t* test.

In order to clarify the potential impact of the *LjLAN* gene on the control of AM symbiosis, the *lan* mutant were inoculated with *Rhizophagus irregularis*. The level of AMF colonization of hyphae and arbuscules of the mutant at 21 dai was significantly lower in comparison with that in WT (Fig 7A–7D). The lower level of AMF colonization was maintained even if the plants were grown for a long time such as 28 and 35 dai following inoculation with *R*. *irregularis* (Fig 7A and 7B). In the *lan* mutant, *R*. *irregularis* tended to colonize in the lateral roots rather than primary roots (Fig 7E). The introduction of WT *LjLAN* gene into the mutant by *A*. *rhizogenes*-mediated hairy root transformation rescued the phenotype relevant to AM symbiosis (Fig 7F and 7G). In the hairy root system, although the defects in AM symbiosis was rescued compered with empty vector control, the colonization level was lower than normal root system. This may be due to the difference in root system. Overall, these results suggest that *LjLAN* is required for the establishment of AM symbiosis. *LjLAN* and *LjCYCLOPS* appear to have additive role for the control of AM symbiosis, as the double mutation of *lan* and *cyclops* had an additive effect on the AM symbiosis (S12 Fig). To gain insight into the phenotype of AM symbiosis from marker genes expression, the expression of *LjSbtM1*, *LjRAM1* and *LjPT4* were next investigated. Similar to previous reports [44, 64, 65], the three genes were specifically and strongly activated by AMF infection in WT plants (Fig 8A–8C). In the *lan* mutant, induction levels of *LjSbtM1* and *LjRAM1* were weaker than those in WT, but the *LjPT4* level was largely unaffected (Fig 8A–8C). AMF colonization was normal in the *LORE1*-tagged mutant (S13 Fig). Expression of *LjLAN* itself seemed to be unaffected by AMF infection (S9D Fig).

### The *lan* mutation affects overall plant growth

In addition to the effect on root nodule and AM symbiosis, the *LjLAN* expression in non-symbiotic organs suggested that the role of LjLAN might not be restricted to the control of plant-microbe symbiosis (S9A Fig). We then examined the effect of the *lan* mutation on shoot and root growth by growing the plants in the soil that contained enough nutrients in the absence of rhizobia and AMF. The shoot and primary root lengths in the *lan* mutant was shorter than WT (S14A–S14C Fig). In addition, shoot branching tended to be promoted in the mutant (S14A Fig). These results suggest that LjLAN has a role in the control of overall plant development. The shoot and root phenotypes of *LORE1*-tagged mutant was indistinguishable from WT plants (S15 Fig).

## Discussion

### Potential molecular function of LjLAN

Mediator is a multiprotein complex that has a fundamental role as an integrator of gene transcription, and governs diverse regulatory processes in plants including development, phytohormone signaling, and responses to biotic and abiotic stress [57–59]. The involvement of Mediator complex in such pleiotropic aspects seems to be achieved by assigning respective Mediator subunits specific functions. In this study, we showed that a nodulation-deficiency phenotype was caused by the mutation of a gene encoding a protein putatively homologous to AtMED2/29/32 subunit of Mediator complex. AtMED2 is required for the recruitment of RNA polymerase II, indicating that AtMED2 has an actual component of the complex [60]. We also demonstrated that AtMED2 could rescue the *lan* mutation. Thus, the functions of LjLAN and AtMED2 seem to be conserved. To the best of our knowledge, this is the first report describing the identification of a Mediator subunit that is involved in plant-microbe symbiosis. Mediator complex subunits are arranged into four modules; the head, middle and tail modules form the core part of Mediator complex, and the kinase module is separable. AtMED2/29/32 is considered as a tail module-type Mediator subunit. The function of AtMED2/29/32 appears to be pleiotropic and it has a role in abiotic stress signaling related to cold and redox, and phenylpropanoid biosynthesis [60, 66, 67]. Arabidopsis MED25/PHYTOCHROME AND FLOWERING TIME 1 (PFT1), which is a member of the tail module, is one of the best characterized Mediator subunits. AtMED25/PFT1 mediates pleiotropic phenomena, including flower and root development, jasmonate signaling, and salinity and water stress by interacting with key TFs acting in specific regulatory processes [58, 68]. Upon stress or developmental stimuli, plants synthesize jasmonate isoleucine, which enables interaction between AtMED25/PFT1 and AtMYC TFs, achieving transcription of jasmonate-responsive genes [69]. In auxin signaling a compositional change in Mediator complex, that includes AtMED13 and AtMED25, upon auxin stimuli enables Arabidopsis AUXIN RESPONSE FACTOR 7 (AtARF7) and AtARF19 to activate expression of downstream genes [70].

As LjLAN is a putative orthologue of AtMED2/29/32, an expected molecular function of LjLAN may be related to mediate gene transcription through interactions predominantly with TF in response to an environmental cue. Then what kind of TF and environmental cue can be involved in this machinery? To date, studies using *L*. *japonicus* and *M*. *truncatula* have identified several TFs involved in nodulation, such as LjCYCLOPS/MtIPD3, Lj/MtNIN, Lj/MtNF-Y subunits, Lj/MtNODULATION SIGNALING PATHWAY 1/2 and Lj/MtERN1/2 [3, 37, 71, 72]. However, the largely severe nodulation phenotype of mutants of these TFs, does not resemble the *lan* nodulation phenotype, although we cannot rue out the possibility that relatively milder *lan* nodulation phenotype may be explained by partial functional redundancy of other Mediator subunits with LjLAN. The arrested nodulation phenotype of *cyclops* is partly similar to the *lan* nodulation phenotype [63], but the analysis of *lan cyclops* double mutant suggests that LjLAN and LjCYCLOPS act in a parallel rather than in a same genetic pathway. Given the normal calcium spiking in the *lan* mutant, LjLAN-mediated transcriptional machinery may act downstream of calcium signaling in parallel with CSP pathway including LjCYCLOPS for the control of nodulation-related gene expression (S16 Fig). Thus, the data so far obtained suggest that LjLAN may interact with unidentified TF(s) rather than known ones. However, we cannot rule out the possibility that *lan* phenotype is due to overall low transcription of key symbiotic genes. An identification of interacting proteins of LjLAN based on the analysis of protein-protein interactions will be undoubtedly needed to verify the possibilities. With respect to the potential environmental cue in this machinery, it seems reasonable to propose that rhizobia infection may be a preferential cue. As the pattern of symbiotic calcium spiking is normal in the *lan* mutant, a more specific cue may be produced downstream of this signal. In an example of a plant-pathogen interaction, oomycete downy mildew pathogen can attenuate salicylic acid-triggered immunity in Arabidopsis by imposing the interaction between its effector and AtMED19a [73]. Hence, it is possible that a rhizobia-derived factor may directly affect plant Mediator complex to control plant gene transcription relevant to nodulation.

As described above, the Mediator complex is involved in different aspects of plant development and environmental responses. Although in this study we put particular emphasis on the role of LjLAN in plant-microbe symbiosis, it is possible that LjLAN is involved in overall plant development because shoot and root growth were affected by the *lan* mutation under nutrient sufficient conditions. In *L*. *japonicus* stable transformation, we use an *A*. *tumefaciens*-medited transformation, where tissue cultures undergo callus formation and shoot regeneration processes. While we were successful in making the transgenic plants with deletion in C-terminal part of LjLAN, we failed to create complete knockout plants of *lan* by aiming to mutate N-terminal part of LjLAN. In addition, in a stable transformation to complement non-symbiotic phenotype of *lan*, no regenerated plants were obtained. Therefore, based on these findings, we reason that null mutations of LjLAN are likely to affect callus formation and/or shoot regeneration processes. The non-symbiotic phenotype of *lan* may provide an intriguing scenario, where a general component of transcriptional machinery had been recruited to the specific functional context during the evolution of plant-microbe symbiosis. To verify this, detailed molecular function and non-symbiotic role of LjLAN need to be elucidated as an important next study.

### *LjLAN* is required for root-hair ITs-mediated intracellular rhizobia accommodation

In *L*. *japonicus* WT plants, rhizobia enter into roots through the intracellular invasion system, that principally depends on the formation of root-hair ITs. The Nod factor signaling pathway has a crucial role in this process by regulating root-hair ITs formation. Generally, defects in the signaling pathway cause complete loss of root-hair ITs formation that is accompanied by no nodule formation. While *L*. *japonicus* has adopted root-hair ITs-mediated intracellular rhizobia accommodation system, the intercellular invasion system can be used in the case where some nodulation-related factors are mutated [11, 50, 54–56]. For example, in *nfr1 nfr5 symrk spontaneous nodule formation 1* (*snf1*) quadruple mutants, intercellular rhizobial invasion takes place despite apparently no root-hair ITs formation, which leads to the formation of functional nodules [56]. The *snf1* plant is a gain-of-function mutant of LjCCaMK, in which spontaneous cortical cell proliferation occurs [16]. This observation indicates that Nod-factor receptors (LjNFR1/5) and LjSYMRK may not be essential to the intercellular invasion process. Furthermore, proliferating cortical cells may need to preexist in order to allow rhizobia to intercellularly enter into roots. In the *lan* mutant, formation of root-hair ITs is strongly compromised, but it is likely that rhizobia can intercellularly enter into roots, as functional nodules are formed. As we could not determine if the *lan* mutation affects cortical-ITs formation, it remains unknown how rhizobia are finally released into nodule cells in the mutant. Due to the delay in nitrogen-fixing nodules, the *lan* mutant exhibit growth defects in a nitrogen-depleted condition until they obtain benefit from symbiotic nitrogen fixation. The delayed nodulation phenotype is thought to be a common feature of some *L*. *japonicus* plants, where the intercellular rhizobial invasion is used to accommodate rhizobia in roots [11, 50, 54–56]. Based on the *lan* phenotype, we propose that the predominant role of LjLAN is to initiate swift and efficient production of nitrogen-fixing nodules by promoting root-hair IT-mediated intracellular rhizobial accommodation. In other words, LjLAN may have a role in preventing protracted and less effective nodulation caused by intercellular rhizobial invasion. Analysis of *L*. *japonicus root hairless* mutants indicates that the intercellular invasion system can be adopted in the plants lacking root hairs [54]. It is unlikely that the intercellular invasion phenotype of *lan* is caused by such physical defects, because root hairs are normally formed in the mutant (S14D and S14E Fig).

It is hypothesized that root-hair IT-mediated intracellular invasion is an evolutionarily advanced invasion system, whereas intercellular rhizobial invasion is the basal pathway [56]. Among the various plants that have an ability to perform root nodule symbiosis, it is estimated that 75% of plants use intracellular invasion and the remaining 25% of plants use the root-hair independent intercellular invasion system [74]. Interestingly, a plant such as *Sesbania rostrata* has a dual mode invasion system where both a root hair-independent intercellular invasion and a root hair-dependent invasion can be used, depending on whether the soil is flooded or dry [75]. Future detailed analysis of LjLAN may contribute to our understanding of the genetic basis and the evolution and diversity of the rhizobial invasion system.

### The role of LjLAN in AM symbiosis

In addition to root nodule symbiosis, the phenotype of the *lan* mutant during AM symbiosis suggests that *LjLAN* is also required for symbiosis with AMF. In contrast to the *lan* nodulation phenotype in which formation of functional nodules eventually takes place, the *lan* mutation continues suppressing the establishment of AM symbiosis. As the pattern of symbiotic calcium oscillation was normal in the *lan* mutant, the *lan* mutation seems to affect the progression of AM symbiosis downstream of calcium signaling. The expression of the AM-inducible genes, *LjSbtM1*, *LjRAM1* and *LjPT4* is generally suppressed by mutation of the CSP genes so far identified [44]. In contrast, while the induction levels of *LjSbtM1* and *LjRAM1* are reduced by the *lan* mutation, that of *LjPT4* is largely unaffected. Currently, it remains almost completely unknown why these genes show different expression patterns in the *lan* mutant. However, based on the loss-of-function phenotypes of each gene, *LjSbtM1* and *LjRAM1* are required for initiation and/or growth of arbuscules [44, 64]. In contrast, a major role of legume *PT4* seems to be associated with phosphate transport [76]. It is unclear if *PT4* is directly involved in arbuscules development. Such differences in the molecular function of three genes might underlie different gene expression patterns depending on the context; while canonical CSP pathway regulates both arbuscules developmental program and phosphate transport by inducing the three genes, the LjLAN-mediated pathway may only regulate arbuscules developmental program by inducing *LjSbtM1* and *LjRAM1*.

AMF accommodation can employ both the intercellular and intracellular dual invasion system [77]. A specialized structure called the prepenetration apparatus (PPA) mediates the intracellular invasion of AMF and has been suggested to share structural similarities with IT [78–80]. Given that LjLAN has a conserved role between root nodule and AM symbiosis, the predominant role of LjLAN in IT formation indicates that LjLAN also may be involved in intracellular AMF accommodation by mediating PPA formation. Future studies investigating the role of LjLAN during AM symbiosis should place particular emphasis on investigating if PPA formation is involved in AMF accommodation. Because of the lack of evidences, it is currently difficult to integratedly interpret the molecular function of LjLAN in root nodule and AM symbiosis. However, based on *lan* phenotype, LjLAN-mediated transcriptional regulatory system could be associated with regulation of genes acting symbiont infection processes. As cell cycle activation such as nuclear enlargement and endreduplication commonly occurs during both symbiont infections, the genes involved in this process may be target genes of LjLAN-mediated regulatory system.

## Materials and methods

### Plant materials and growth conditions

The Miyakojima MG-20 and Gifu B-129 ecotype of *L*. *japonicus* was used as the WT in this study. The *lan* mutant was isolated as a result of screen for nodulation-deficient mutants using the M_2_ generation of WT plants that had been mutagenized with 0.4% ethylmethane sulfonate (EMS) for 6 hours. The *LORE1*-tagged line of *lan* (Plant ID: 30008618) was obtained from Lotus Base (https://lotus.au.dk). A description of the *DR5*:*GFP-NLS* plants and *cyclops-6* has been published previously [45]. *ccamk-14* mutant with MG-20 background was newly identified in this study. For the analysis of root nodule symbiosis, plants were grown with or without *M*. *loti* MAFF 303099 as previously described [81]. *M*. *loti nodC* mutant was obtained from LegumeBase (https://www.legumebase.brc.miyazaki-u.ac.jp/top.jsp). For the analysis of the AM symbiosis, plants were grown with or without *R*. *irregularis* (DAOM197198; PremierTech) as previously described [65].

### Acetylene reduction assay

The nitrogenase activity of nodules was indirectly determined by measuring acetylene reductase activity (nmol/ h per plant) as previously described [82].

### Genome-resequencing of the *lan* mutant

The leaves of the *lan* mutant were ground with liquid nitrogen using a mortar and pestle. Genomic DNA was isolated using a DNeasy Plant Mini Kit (Qiagen). The quality of purified genomic DNA was evaluated by a Quant-iT dsDNA BR Assay Kit (Invitrogen). For whole-genome shotgun sequencing of the *lan* mutant, we performed paired-end sequencing with HiSeq 2000 (Illumina). After fragmentation of the isolated genomic DNA, an Illumina library with a mean insertion length of 350-bp was constructed using TruSeq Nano DNA LT Sample Preparation Kit (Illumina) following the manufacturer’s instructions. These libraries were subsequently sequenced 101 bp from both ends, yielding 6.25 gigabase (Gb) of raw data. After the removal of adaptor sequences and low quality reads (Phred quality score ≥ 20 in < 90% of the bases), 5.97 Gb of high quality sequences remained. The remaining reads were mapped against *L*. *japonicus* genome assembly build 2.5 using the Bowtie software [83]. The median value of per-base sequence depth was 18.3 and the genome coverage was 90.2%. The resulting data in the sam format were converted into bam format using Samtools [84]. Genome-wide SNPs were called from the bam files using Samtools and Bedtools [85]. A SNP that is specific to the *lan* mutant was found by examining the mapped region harboring the *LjLAN* locus with Integrative Genomics Viewer program (https://www.broadinstitute.org/igv/).

### Constructs, hairy-root and stable transformation of *L*. *japonicus*

The primers used for PCR are listed in S1 Table. For the complementation analysis, a 5.8-kb genomic DNA fragment including the *LjLAN* candidate gene was amplified by PCR from WT genomic DNA. This fragment including 4.4 kb of sequence directly upstream of the initiation codon, was cloned into pCAMBIA1300-GFP-LjLTI6b [45]. The coding sequences (cds) of *LjLAN* and *LjLAN*Δ*C* were, respectively, amplified by PCR from template cDNA prepared from WT *L*. *japonicus*. The cds of *AtMED2* was amplified by PCR from template cDNA prepared from Arabidopsis Col-0 plants. They were cloned into the pENTR/D-TOPO vector (Invitrogen). The insert was transferred into pUB-GW-GFP [86] by the LR recombination reaction. To obtain the *ProLjLAN*:*GUS plus* construct, first an artificially-synthesized *GUS plus* gene was cloned into pENTR/D-TOPO vector to create the vector pENTR-gus plus. The *GUS plus* gene in pENTR-gus plus was introduced into a vector pCAMBIA1300-GW-GFP-LjLTI6b [87] by the LR recombination reaction to create the vector pCAMBIA1300-GUS plus-GFP-LjLTI6b. Next, 4.4 kb of sequence directly upstream of the initiation codon of *LjLAN* was amplified by PCR and cloned upstream of *GUS plus* gene of pCAMBIA1300-GUS plus-GFP-LjLTI6b to create the vector pCAMBIA1300-pLjLAN-GUS plus-GFP-LjLTI6b. For the analysis of calcium spiking, we used a construct in which nuclear-localized yellow-chameleon (YC2.60) was expressed under the control of the *LjUBQ* promoter [81]. The recombinant plasmids were introduced into *A*. *rhizogenes* strain AR1193 [88] and were transformed into roots of *L*. *japonicus* plants by a hairy-root transformation method as previously described [45].

To create CRISPR-Cas9 construct of *LjLAN*, targeting site in the gene was designed using the CRISPR-P program (http://cbi.hzau.edu.cn/crispr/) [89]. Oligonucleotide pairs (S1 Table) were annealed and cloned into a single guide RNA (sgRNA) cloning vector, pUC19_AtU6oligo, as previously described [90]. Then, the sgRNA expression cassette prepared in pUC19_AtU6oligo was excised and replaced with OsU3:gYSA in pZH_gYSA_FFCas9, an all-in-one binary vector harboring a sgRNA, Cas9, and an HPT expression construct, as previously described [90]. The recombinant plasmid was introduced into *A*. *tumefaciens* strain AGL1 and was transformed into WT *L*. *japonicus* MG-20 plants by a stable transformation method as previously described [82].

### Expression analysis

The primers used for PCR are listed in S1 Table. Total RNA was isolated from each plant tissue using the RNeasy Plant Mini Kit (Qiagen) or the PureLink Plant RNA Reagent (Invitrogen). First-strand cDNA was prepared using the ReverTra Ace qPCR RT Master Mix with gDNA Remover (Toyobo). Real-time RT-PCR was performed using a Light Cycler 96 System (Roche) or a 7900HT Real-Time PCR system (Applied Biosystems) with a THUNDERBIRD SYBR qPCR Mix (Toyobo) according to the manufacturer’s protocol. The expression of *LjUBQ* was used as the reference. Data are shown as mean±SD of 3–4 biological replicates.

### Accession number

Sequence data from this article can be found in the GenBank/EMBL data libraries under the following accession numbers: LjLAN, LC171403; LjLAN LIKE, LC194237. Data of short reads from the *lan* genomic DNA has been deposited in the DNA Data Bank of Japan Sequence Read Archive under the accession number DRA004948.

## Supporting information

S1 Table Primers used in this work. (XLS)

S1 Fig *lan* nodulation phenotype of later nodulation stages and inoculation of *M. loti nodC* mutants. (A) The numbers of sites of cortical cell proliferation (CCP) and of nodule primordia (NP), and mature nodules in *DR5*:*GFP-NLS*/WT MG-20 and in the *DR5*:*GFP-NLS*/*lan* plants at 45 dai (n = 11 plants). CCP was identified by *GFP-NLS* signals that were expressed under the control of *DR5*. When cortical cells appeared bulged by the progress of several rounds of cell division, the sites were judged as NP. Mature nodules were judged by several indicators including sizes, colors and lenticels formation. Student’s t-test was performed by comparing total nodule number. *P < 0.05 by Student’s *t* test. (B) Average nodule number in the WT MG-20 and *lan* inoculated with *M*. *loti* WT or *nodC* mutants at 21 dai (n = 9–12 plants).
(TIF)

S2 Fig Patterns of calcium spiking. Transgenic hairy roots containing the nuclear-localized yellow-chameleon (YC2.60) construct were analyzed. Nod-factor (A and B) or water (C and D) were applied to WT MG-20 (A and C) and *lan* (B and D) roots. In this experimental condition, Nod factor treatment generated calcium spiking in 14/48 WT MG-20 and 29/104 *lan* root cells, whereas water treatment generated no calcium spiking in 0/32 WT MG-20 and 0/24 *lan* root cells. Representative calcium spiking pattern for 30 min. are shown for each genotype.
(TIF)

S3 Fig Close-up images showing effect of the *lan* mutation in early nodule development. (A and B) Nodule primordia formed on 4 dai *DR5*:*GFP-NLS*/WT MG-20 (A) and on 11 dai *DR5*:*GFP-NLS*/*lan* (B) roots. (C and D) Merged images of patterns of rhizobial invasion and auxin response of nodule primordia formed on 4 dai *DR5*:*GFP-NLS*/WT MG-20 (C) and on 11 dai *DR5*:*GFP-NLS*/*lan* (D) roots. *M*. *loti* MAFF303099 constitutively expressing *DsRED* was used for inoculation in A-D. Closed and open arrowheads respectively indicate root-hair ITs and accumulation of rhizobia. Scale bars: 200 μm.
(TIF)

S4 Fig Map-based cloning of *LjLAN*. The *lan* locus was mapped using F_2_ population derived from a cross between *lan* and Gifu B-129 plants. 108 F_2_ plants that exhibited the nodulation-deficient phenotype were used for this analysis. Arrow indicates the *LAN* candidate gene (chr3.CM0112.280.r2.d) found in the *L*. *japonicus* genomic sequence database. The primers used for PCR are listed in S1 Table.
(TIF)

S5 Fig Exon-intron structure of the *LjLAN* gene and the site of mutation in the *lan* mutant. Boxes indicate exons. Initiation codon (ATG) of *LjLAN* is marked in magenta. Splice (GT) and acceptor (AG) site of intron is marked in blue. Thick arrows indicate locations of primer sets used for RT-PCR analysis in Fig 4A. The position of introns in *LjLAN* in WT was determined by sequencing the RT-PCR product. Intron mis-splicing of *LjLAN* in *lan* was determined by sequencing the two RT-PCR products in Fig 4A; minor and major cDNA of *lan* are derived from RT-PCR products with big and small sizes.
(TIF)

S6 Fig *LjLAN* expression pattern. (A) RT-PCR analysis of the *LjLAN* gene. *LjUBQ* was used as the RNA loading control. The locations of primer sets used for PCR is shown in S5 Fig. cDNA was prepared from total RNAs roots (0), and in inoculated roots at 1, 7 and 14 dai. (B) Real-time RT-PCR analysis of *LjLAN* in *lan* and the *LORE1*-tagged line of *lan* (Plant ID: 30008618). cDNA was prepared from total RNAs roots at 7 dai. *LjUBQ* was used to assess the relative expression of the gene. Error bars indicate SD. *P < 0.05 by Student’s *t* test.
(TIF)

S7 Fig LjLAN is a putative orthologue of AtMED2/29/32. (A) Phylogenetic tree of LjLAN-related proteins. Full-length amino acids sequences were compared and the tree was constructed by neighbor-joining methods. Numbers indicate bootstrap values. (B) Amino acid alignment of the LjLAN-related proteins. The amino acid residues with 100% homology among the proteins are shown in white character on a black background. The amino acid residues with 50–85% homology have gray background.
(TIF)

S8 Fig Complementation analysis. (A) Average nodule number in WT and the *lan* mutant with transgenic roots containing respective constructs at 14 dai (n = 17–22 plants). NP, nodule primrdia. (B-F) Representative transgenic hairy roots of WT MG-20 (B) or *lan* (C-F) constitutively expressing *GUS* (B and F), *LjLAN* (C), *AtMED2* (D), *LjLAN*Δ*C* (E) at 14 dai. The detail of LjLANΔC is shown in S11 Fig. Transgenic roots were identified by the expression of GFP. Scale bars: 1 mm. Error bars indicate SD. Columns with the same lower-case letter indicate no significant difference (Tukey’s test, P < 0.05).
(TIF)

S9 Fig Expression patterns of *LjLAN* and *LjLAN LIKE*. (A and B) Real-time RT-PCR analysis of *LjLAN* (A) and *LjLAN LIKE* (B) expression in reproductive and vegetative organs. Each cDNA sample was prepared from total RNA derived from the flower, leaf, stem, shoot apex, non-inoculated (-) and 1 dai (+) roots. (C) Real-time RT-PCR analysis of *LjLAN* expression in uninoculated WT MG-20 (0) and in inoculated roots at 1, 7, 14 dai following inoculation with rhizobia, and in root segments, where proliferating cortical cells were enriched (CCP). cDNAs were prepared from total RNAs from whole roots except for CCP. CCP was prepared by collecting the tissues by the expression of GFP-NLS at 5 dai after *DR5*:*GFP-NLS*/WT MG-20 plants were inoculated with rhizobia. The relative (fold) changes in expression are shown compared to roots at 0 dai. (D) Real-time RT-PCR analysis of *LjLAN* expression in uninoculated (-Myc) and in 21 dai inoculated (+Myc) roots following inoculation with *R*. *irregularis*. cDNAs were prepared from total RNAs from whole roots. *LjUBQ* was used to assess the relative expression of each gene. Error bars indicate SD. *P < 0.05 by Student’s *t* test.
(TIF)

S10 Fig Spatial expression patterns of *LjLAN* during nodulation. (A-E) GUS staining pattern of WT MG-20 transgenic hairy roots containing the *ProLjLAN*:*GUS plus* construct at 4 dai (A-D) and 9 dai (E). Closed and open arrowheads respectively indicate nodulation foci and curled root hair. Arrows indicate lateral roots. Scale bars: 1 mm (A, E); 100 μm (B-D).
(TIF)

S11 Fig Nodulation phenotypes of *LORE1*-tagged *lan* lines and *lan* plants created by the CRISPR/Cas9 genome editing system. (A) A schematic diagram of *LORE1* insertion site in the *LORE1*-tagged line of *lan* (Plant ID: 30008618). (B) Amino acid alignment of several truncated LjLAN used in this study. The amino acids sequence of LjLAN in 30008618 and two *lan* CRISPR lines (*lan #1* and *lan #8*) were determined by sequencing RT-PCR products derived from each plant. The amino acid residues with 100% homology among the proteins are shown in white character on a black background. The amino acid residues with 50–80% homology have gray background. (C and D) Nodulation phenotype of WT Gifu plants and 30008618 at 14 dai. (E) The position of mutations in *lan* plants created by the CRISPR-Cas9 genome editing system. Nucleotide alignment of *LjLAN* is shown. The indel mutations occur near the protospacer adjacent motif (PAM) site (blue letters). The sgRNA target is underlined. (F and G) Nodulation phenotype of WT MG-20 plants, *lan #1* and *lan #8* at 14 dai. For nodulation analysis of *lan #1* and *lan #8*, T2 generation was used, in which respective homozygous mutation was fixed. NP, nodule primordia. Scale bar: 1 mm. Error bars indicate SD. Tukey’s test was performed by comparing total nodule number. Columns with the same lower-case letter indicate no significant difference.
(TIF)

S12 Fig AM symbiosis phenotypes of *lan cyclops* double mutant. (A and B) *R*. *irregularis* colonization ratio of hyphae (A) and arbuscules (B) at 28 dai. (n = 8 plants). Error bars indicate SD. Columns with the same lower-case letter indicate no significant difference (Tukey’s test, P < 0.05).
(TIF)

S13 Fig AM symbiosis phenotypes of *LORE1*-tagged lines of lan. (A and B) *R*. *irregularis* colonization ratio of hyphae (A) and arbuscules (B) at 21 dai. (n = 6 plants). Error bars indicate SD.
(TIF)

S14 Fig The effect of the *lan* mutation on shoot and root growth. (A) WT MG-20 (left) and *lan* (right) plants at 14 days after germination (dag). (B and C) shoot (B) and primary root (C) length at 14 dag. Plants were grown in the soil that contained enough nutrients in the absence of rhizobia and AMF. (D and E) Root hairs phenotype of WT MG-20 (D) and *lan* (E) grown on agar plate at 3 dag. Scale bar: 1 cm. Error bars indicate SD. *P < 0.05 by Student’s *t* test.
(TIF)

S15 Fig Shoot and root growth of *LORE1*-tagged lines of *lan*. (A) WT Gifu (left) and 30008618 (right) plants at 14 days after germination (dag). (B and C) shoot (B) and primary root (C) length at 14 dag. Plants were grown in the soil that contained enough nutrients in the absence of rhizobia and AMF. Scale bar: 1 cm. Error bars indicate SD.
(TIF)

S16 Fig Model for the position of LjLAN-mediated regulation in root nodule symbiosis. Perception of rhizobia-derived Nod factor by its receptors elicits Nod factor signaling. Consequently, calcium signaling is induced, which is decoded by LjCCaMK. LjCCaMK then activates LjCYCLOPS, which directly induces *LjNIN* expression. As normal calcium spiking pattern was observed in the *lan* mutant, the expected position of LjLAN-mediated regulation may be downstream of LjCCaMK. Nodulation phenotype and *LjNIN* expression in *lan cyclops* double mutant suggest that LjLAN and LjCYCLOPS act in parallel for the regulation of *LjNIN* expression. LjLAN, a subunit of Mediator complex, can achieve the regulation by interacting with unidentified transcription factor (TF).
(TIF)
