# Correction: *LACK OF SYMBIONT ACCOMMODATION* controls intracellular symbiont accommodation in root nodule and arbuscular mycorrhizal symbiosis in *Lotus japonicus*

**DOI:** 10.1371/journal.pgen.1007966

**Published:** 2019-01-31

**Authors:** Takuya Suzaki, Naoya Takeda, Hanna Nishida, Motomi Hoshino, Momoyo Ito, Fumika Misawa, Yoshihiro Handa, Kenji Miura, Masayoshi Kawaguchi

## Notice of republication

This article was republished on January 14, 2019, to correct an error in the title: the species name *Lotus japonicus* was misspelled as *Lotus japonius*. Please download this article again to view the correct version.
