# Supplementary figures and images for: LACK OF SYMBIONT ACCOMMODATION controls intracellular symbiont accommodation in root nodule and arbuscular mycorrhizal symbiosis in Lotus japonicus
Source: PLoS Genet. 2019 Jan 3;15(1):e1007865. doi: 10.1371/journal.pgen.1007865 (PMC6317779; doi:10.1371/journal.pgen.1007865)

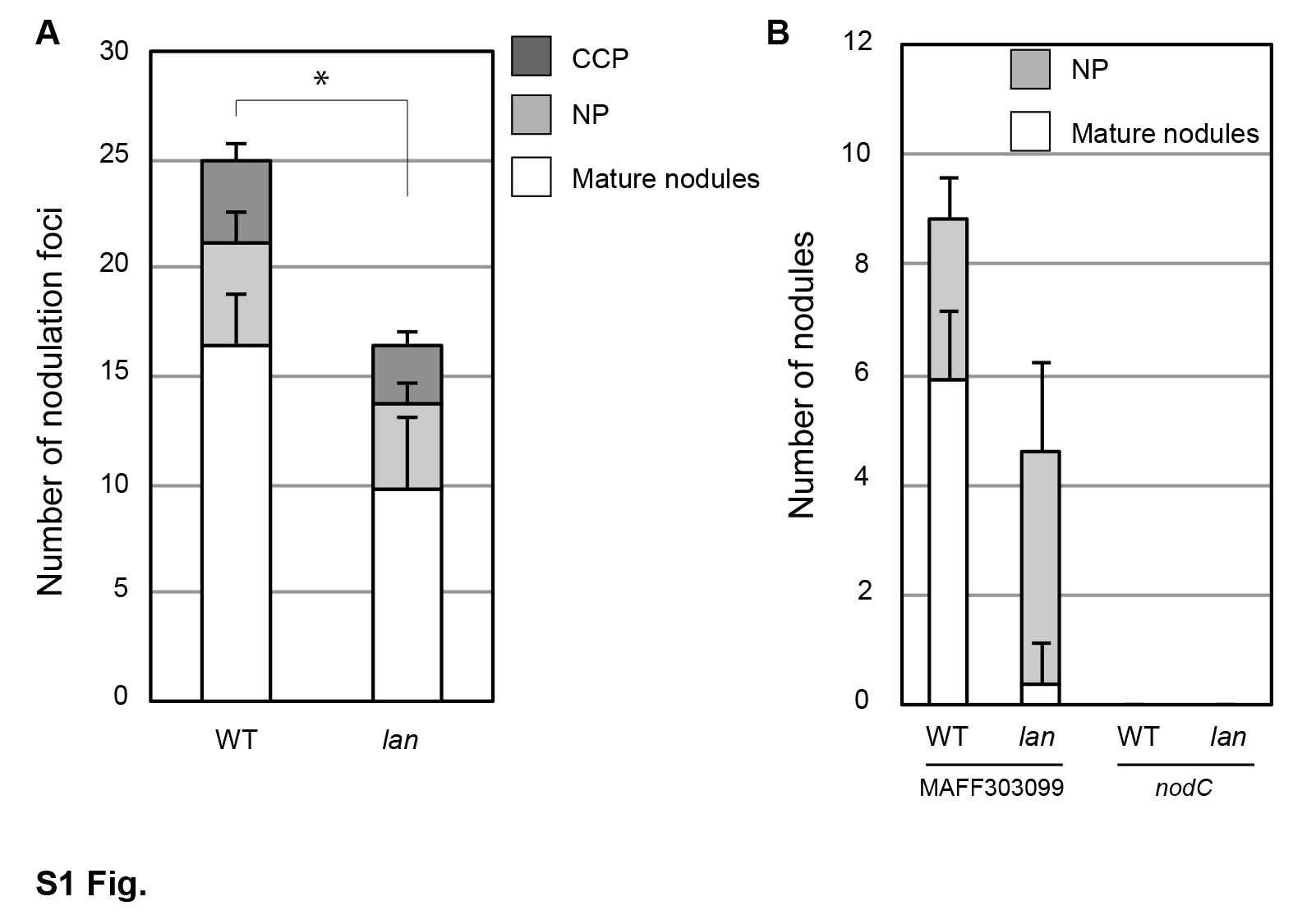

Supplement: S1 Fig — (A) The numbers of sites of cortical cell proliferation (CCP) and of nodule primordia (NP), and mature nodules in DR5:GFP-NLS/WT MG-20 and in the DR5:GFP-NLS/lan plants at 45 dai (n = 11 plants). CCP was identified by GFP-NLS signals that were expressed under the control of DR5. When cortical cells appeared bulged by the progress of several rounds of cell division, the sites were judged as NP. Mature nodules were judged by several indicators including sizes, colors and lenticels formation. Student’s t-test was performed by comparing total nodule number. *P < 0.05 by Student’s t test. (B) Average nodule number in the WT MG-20 and lan inoculated with M. loti WT or nodC mutants at 21 dai (n = 9–12 plants). (TIF) [file pgen.1007865.s002.tif]

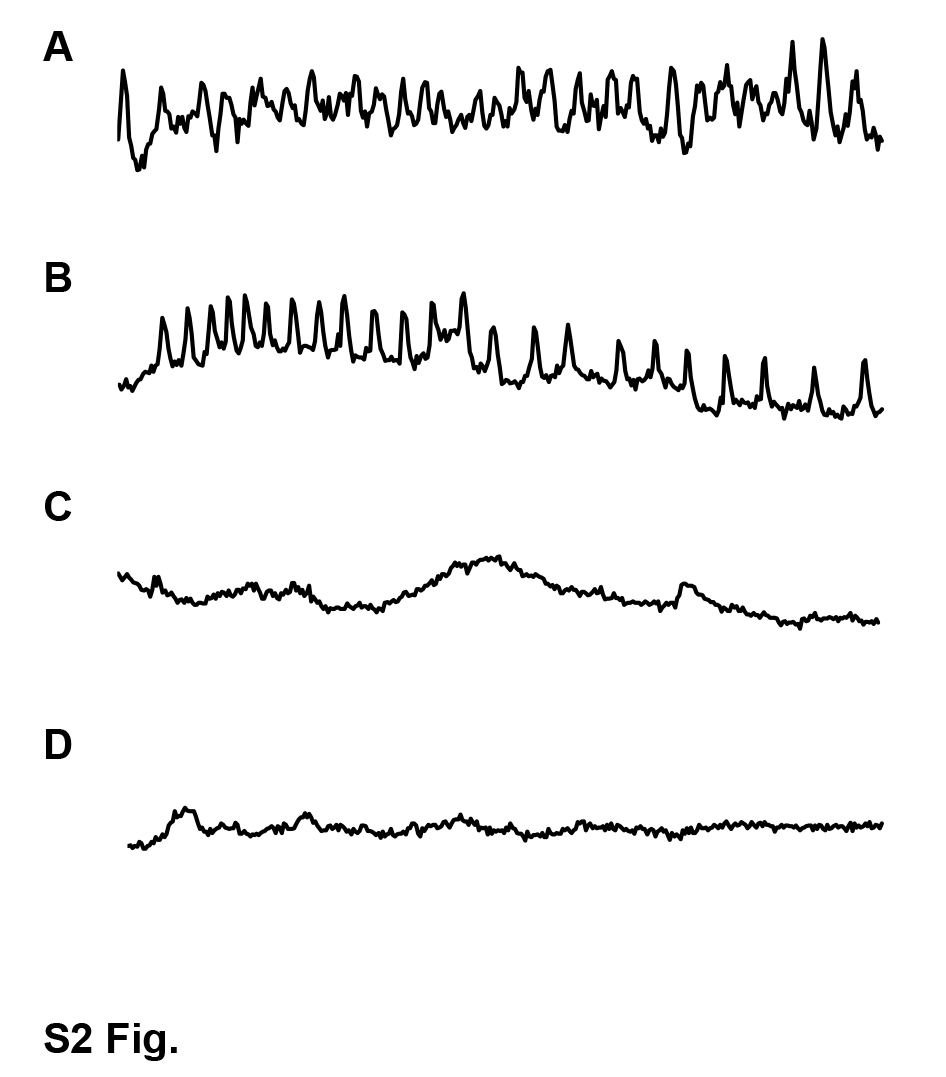

Supplement: S2 Fig — Transgenic hairy roots containing the nuclear-localized yellow-chameleon (YC2.60) construct were analyzed. Nod-factor (A and B) or water (C and D) were applied to WT MG-20 (A and C) and lan (B and D) roots. In this experimental condition, Nod factor treatment generated calcium spiking in 14/48 WT MG-20 and 29/104 lan root cells, whereas water treatment generated no calcium spiking in 0/32 WT MG-20 and 0/24 lan root cells. Representative calcium spiking pattern for 30 min. are shown for each genotype. (TIF) [file pgen.1007865.s003.tif]

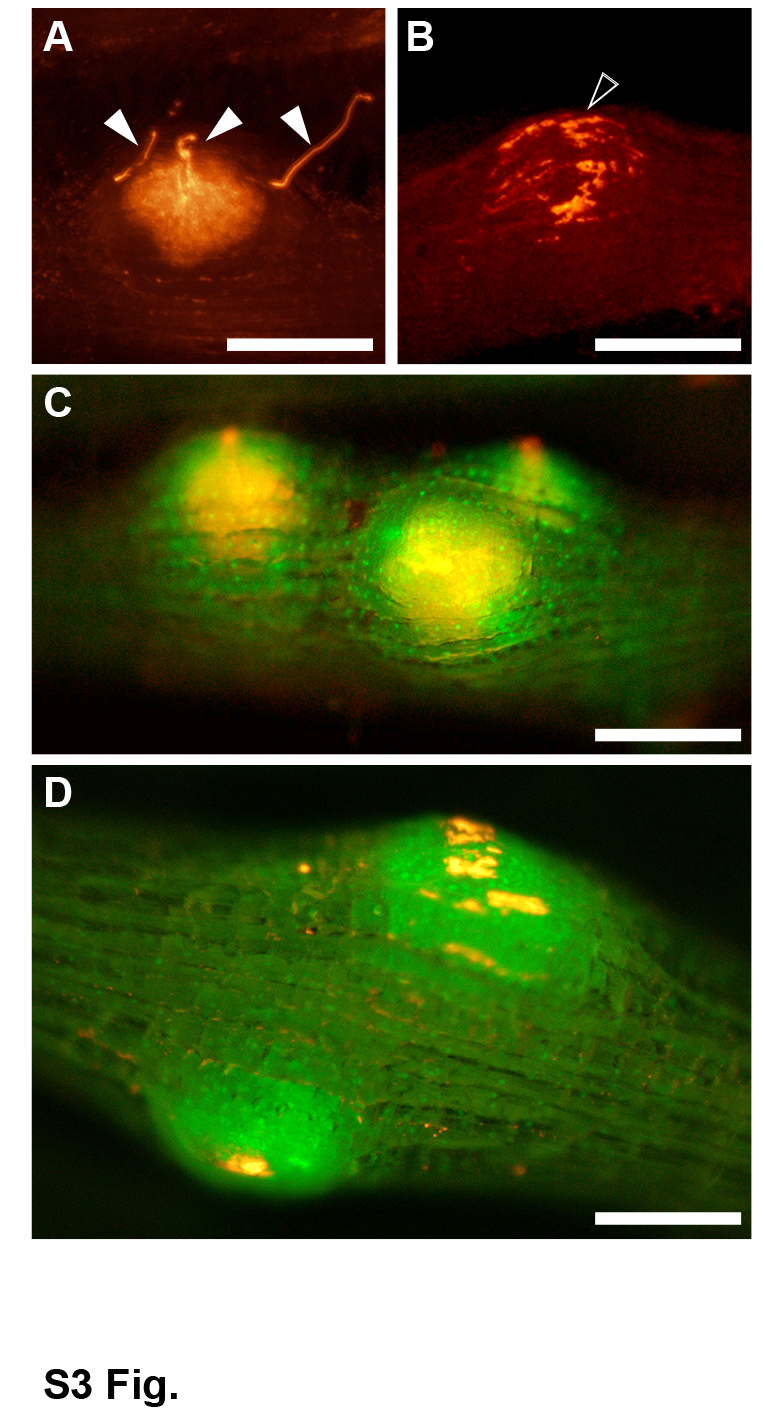

Supplement: S3 Fig — (A and B) Nodule primordia formed on 4 dai DR5:GFP-NLS/WT MG-20 (A) and on 11 dai DR5:GFP-NLS/lan (B) roots. (C and D) Merged images of patterns of rhizobial invasion and auxin response of nodule primordia formed on 4 dai DR5:GFP-NLS/WT MG-20 (C) and on 11 dai DR5:GFP-NLS/lan (D) roots. M. loti MAFF303099 constitutively expressing DsRED was used for inoculation in A-D. Closed and open arrowheads respectively indicate root-hair ITs and accumulation of rhizobia. Scale bars: 200 μm. (TIF) [file pgen.1007865.s004.tif]

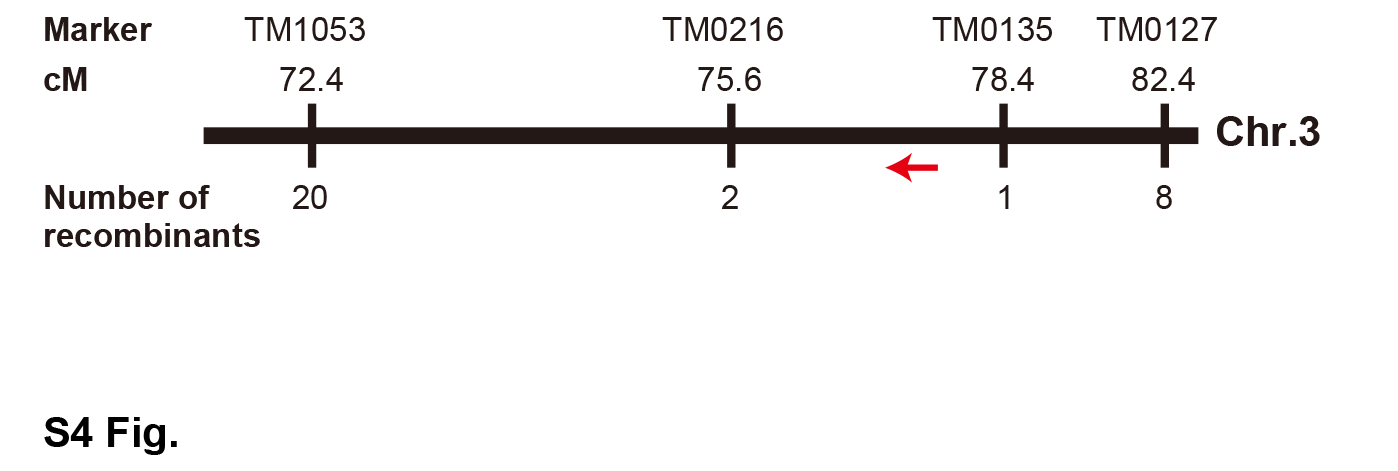

Supplement: S4 Fig — The lan locus was mapped using F2 population derived from a cross between lan and Gifu B-129 plants. 108 F2 plants that exhibited the nodulation-deficient phenotype were used for this analysis. Arrow indicates the LAN candidate gene (chr3.CM0112.280.r2.d) found in the L. japonicus genomic sequence database. The primers used for PCR are listed in S1 Table. (TIF) [file pgen.1007865.s005.tif]

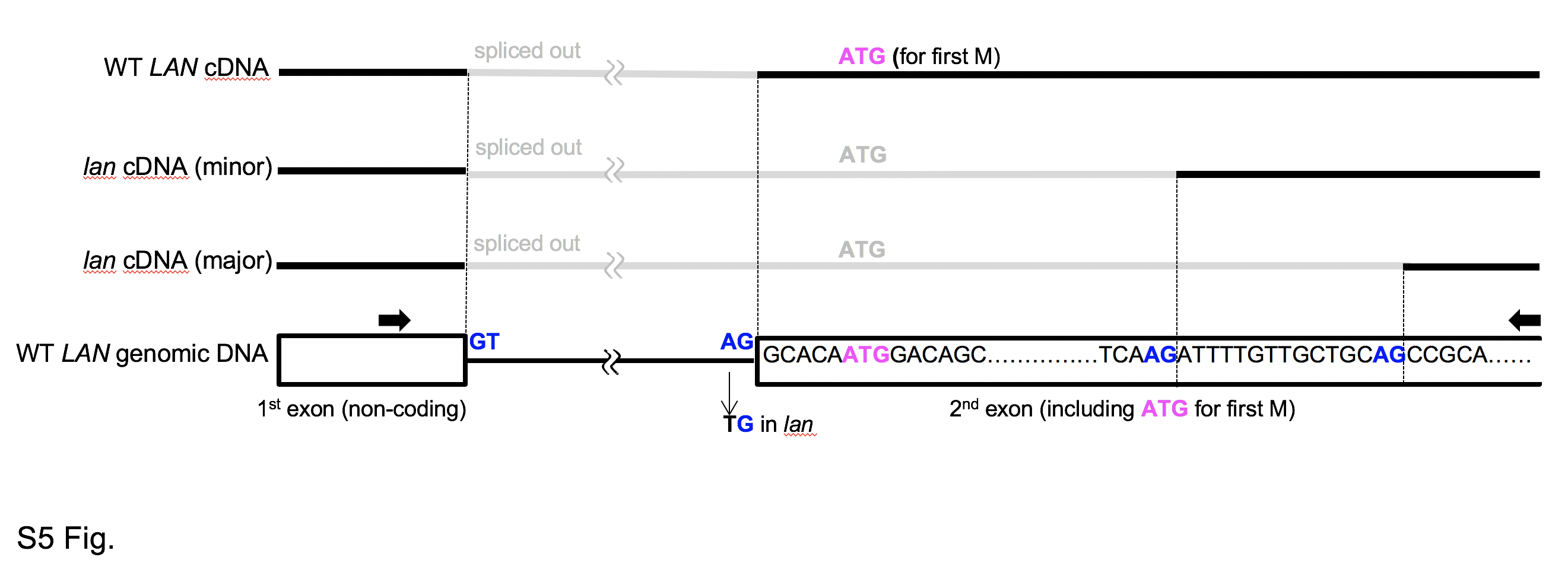

Supplement: S5 Fig — Boxes indicate exons. Initiation codon (ATG) of LjLAN is marked in magenta. Splice (GT) and acceptor (AG) site of intron is marked in blue. Thick arrows indicate locations of primer sets used for RT-PCR analysis in Fig 4A. The position of introns in LjLAN in WT was determined by sequencing the RT-PCR product. Intron mis-splicing of LjLAN in lan was determined by sequencing the two RT-PCR products in Fig 4A; minor and major cDNA of lan are derived from RT-PCR products with big and small sizes. (TIF) [file pgen.1007865.s006.tif]

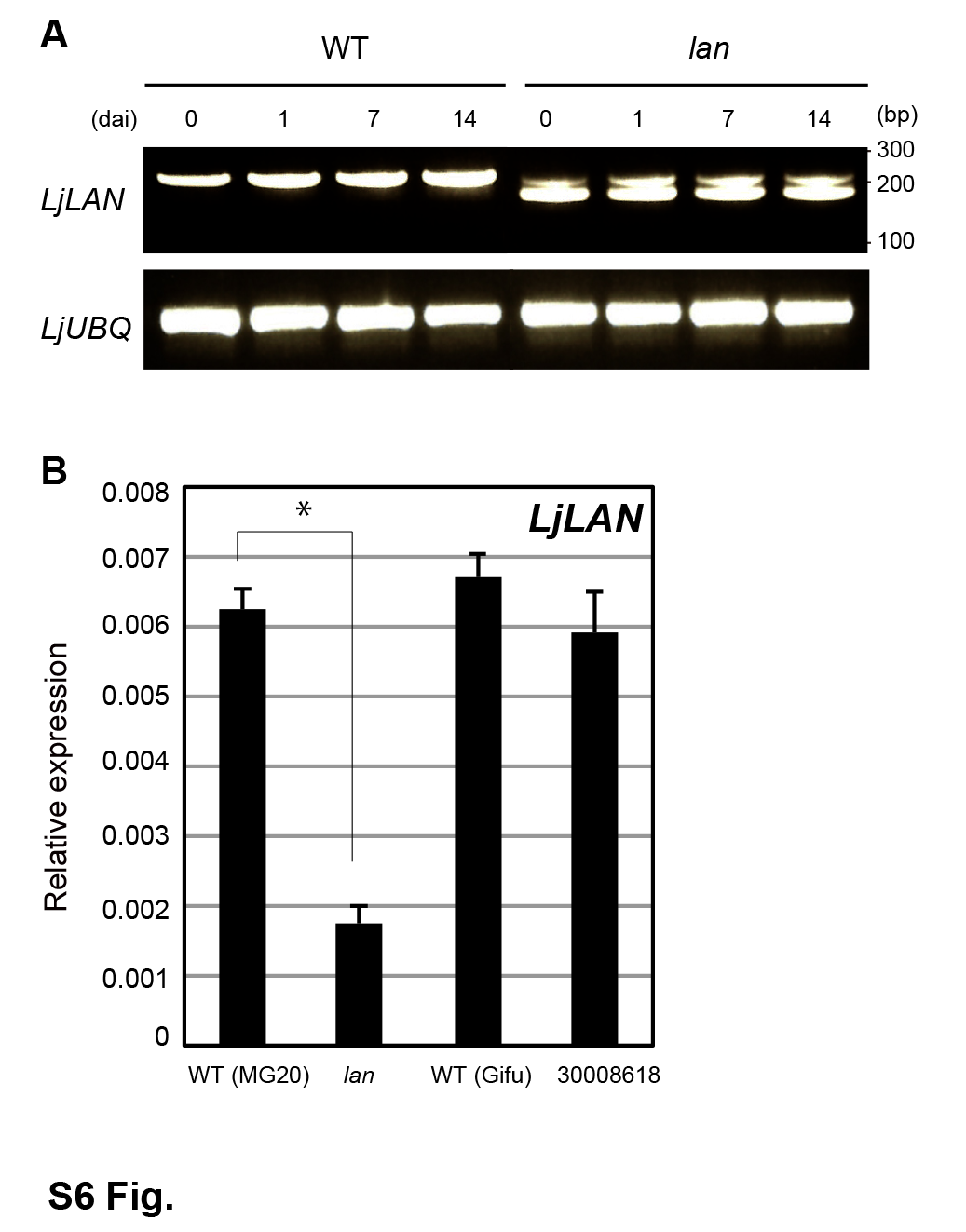

Supplement: S6 Fig — (A) RT-PCR analysis of the LjLAN gene. LjUBQ was used as the RNA loading control. The locations of primer sets used for PCR is shown in S5 Fig. cDNA was prepared from total RNAs roots (0), and in inoculated roots at 1, 7 and 14 dai. (B) Real-time RT-PCR analysis of LjLAN in lan and the LORE1-tagged line of lan (Plant ID: 30008618). cDNA was prepared from total RNAs roots at 7 dai. LjUBQ was used to assess the relative expression of the gene. Error bars indicate SD. *P < 0.05 by Student’s t test. (TIF) [file pgen.1007865.s007.tif]

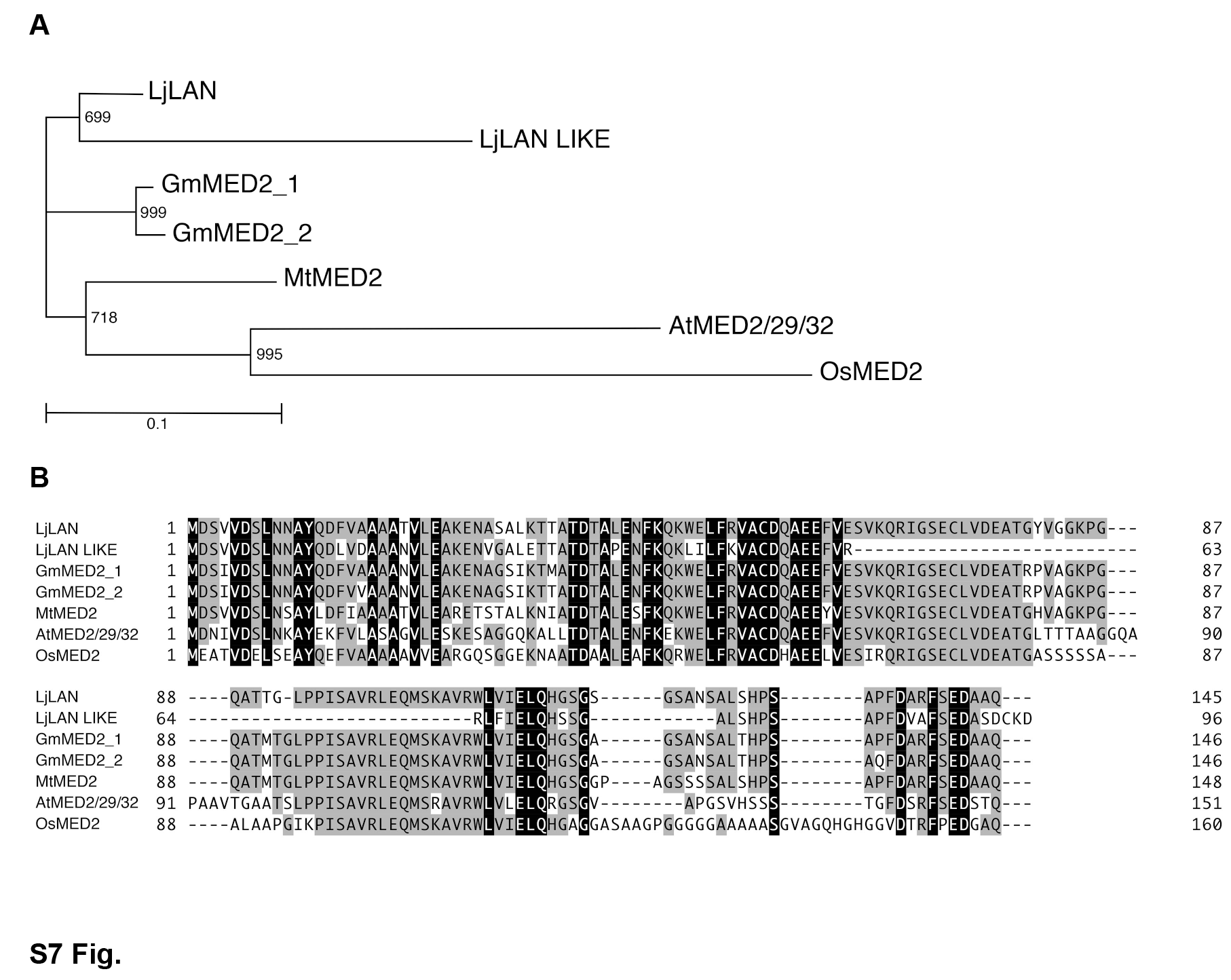

Supplement: S7 Fig — (A) Phylogenetic tree of LjLAN-related proteins. Full-length amino acids sequences were compared and the tree was constructed by neighbor-joining methods. Numbers indicate bootstrap values. (B) Amino acid alignment of the LjLAN-related proteins. The amino acid residues with 100% homology among the proteins are shown in white character on a black background. The amino acid residues with 50–85% homology have gray background. (TIF) [file pgen.1007865.s008.tif]

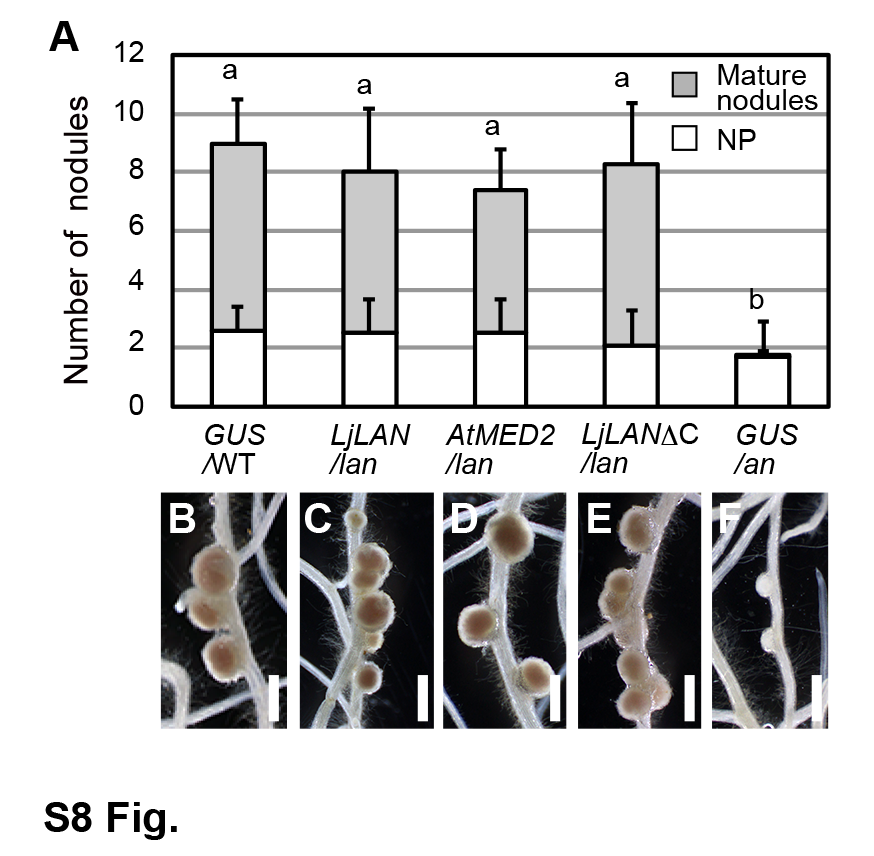

Supplement: S8 Fig — (A) Average nodule number in WT and the lan mutant with transgenic roots containing respective constructs at 14 dai (n = 17–22 plants). NP, nodule primrdia. (B-F) Representative transgenic hairy roots of WT MG-20 (B) or lan (C-F) constitutively expressing GUS (B and F), LjLAN (C), AtMED2 (D), LjLANΔC (E) at 14 dai. The detail of LjLANΔC is shown in S11 Fig. Transgenic roots were identified by the expression of GFP. Scale bars: 1 mm. Error bars indicate SD. Columns with the same lower-case letter indicate no significant difference (Tukey’s test, P < 0.05). (TIF) [file pgen.1007865.s009.tif]

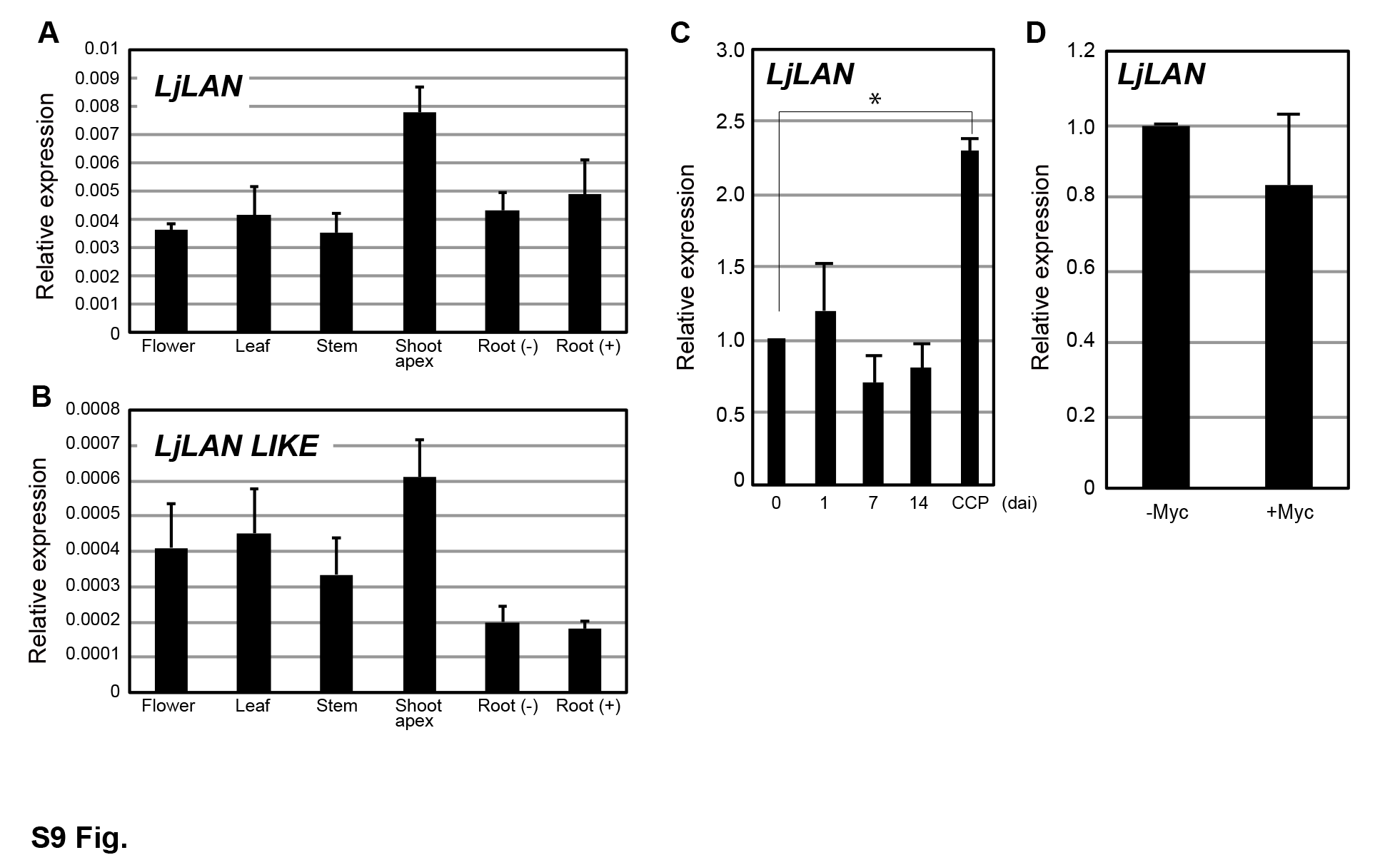

Supplement: S9 Fig — (A and B) Real-time RT-PCR analysis of LjLAN (A) and LjLAN LIKE (B) expression in reproductive and vegetative organs. Each cDNA sample was prepared from total RNA derived from the flower, leaf, stem, shoot apex, non-inoculated (-) and 1 dai (+) roots. (C) Real-time RT-PCR analysis of LjLAN expression in uninoculated WT MG-20 (0) and in inoculated roots at 1, 7, 14 dai following inoculation with rhizobia, and in root segments, where proliferating cortical cells were enriched (CCP). cDNAs were prepared from total RNAs from whole roots except for CCP. CCP was prepared by collecting the tissues by the expression of GFP-NLS at 5 dai after DR5:GFP-NLS/WT MG-20 plants were inoculated with rhizobia. The relative (fold) changes in expression are shown compared to roots at 0 dai. (D) Real-time RT-PCR analysis of LjLAN expression in uninoculated (-Myc) and in 21 dai inoculated (+Myc) roots following inoculation with R. irregularis. cDNAs were prepared from total RNAs from whole roots. LjUBQ was used to assess the relative expression of each gene. Error bars indicate SD. *P < 0.05 by Student’s t test. (TIF) [file pgen.1007865.s010.tif]

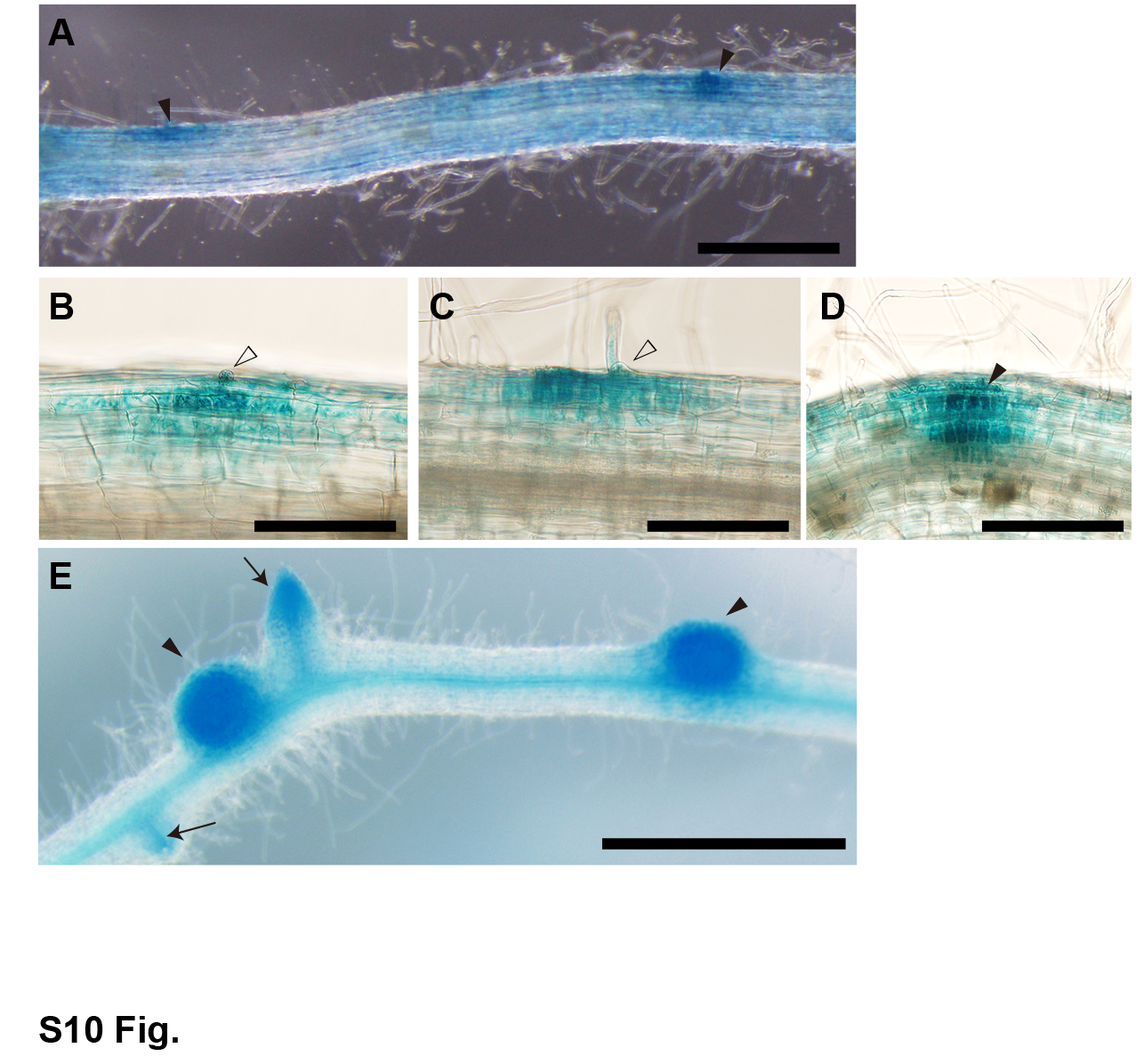

Supplement: S10 Fig — (A-E) GUS staining pattern of WT MG-20 transgenic hairy roots containing the ProLjLAN:GUS plus construct at 4 dai (A-D) and 9 dai (E). Closed and open arrowheads respectively indicate nodulation foci and curled root hair. Arrows indicate lateral roots. Scale bars: 1 mm (A, E); 100 μm (B-D). (TIF) [file pgen.1007865.s011.tif]

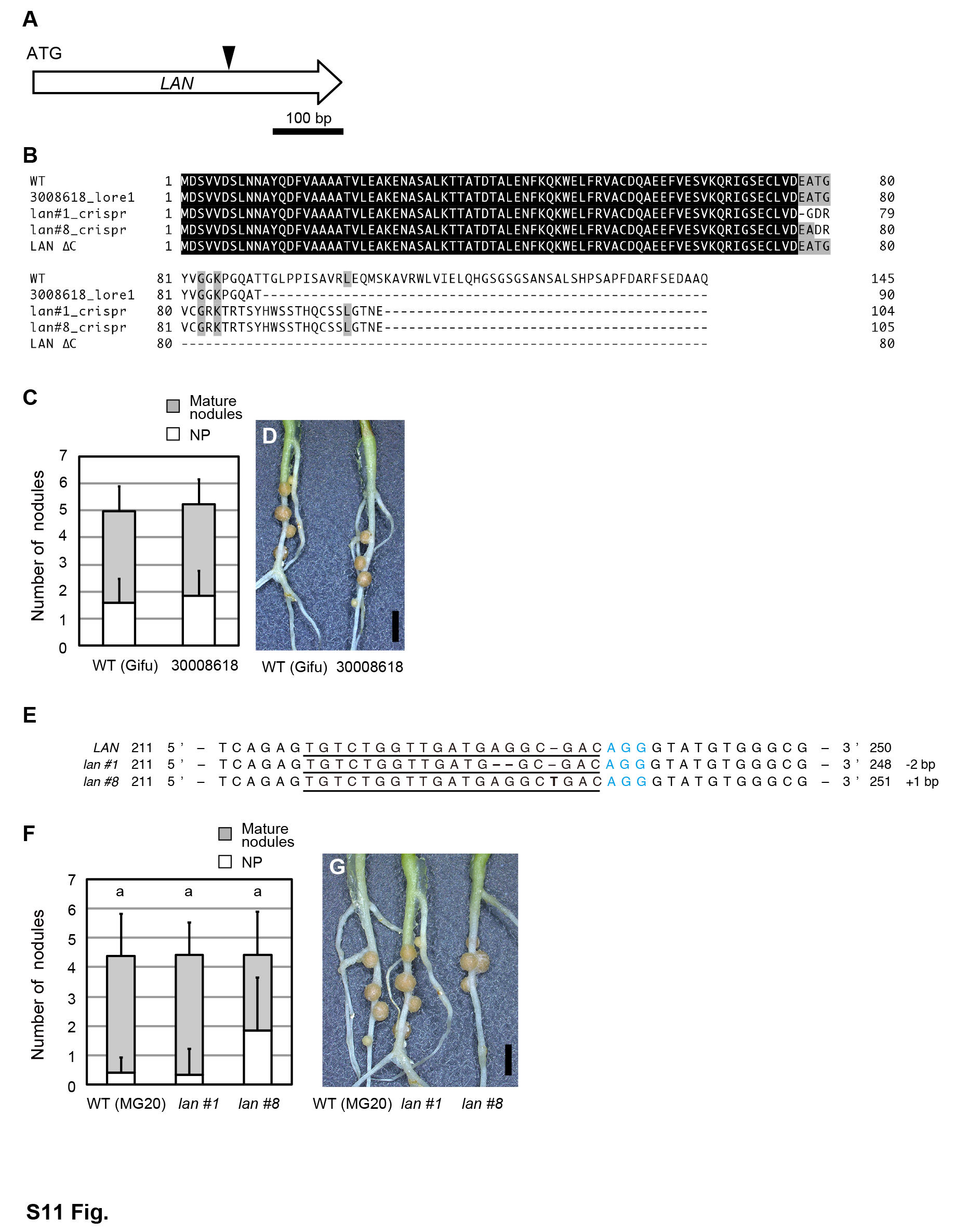

Supplement: S11 Fig — (A) A schematic diagram of LORE1 insertion site in the LORE1-tagged line of lan (Plant ID: 30008618). (B) Amino acid alignment of several truncated LjLAN used in this study. The amino acids sequence of LjLAN in 30008618 and two lan CRISPR lines (lan #1 and lan #8) were determined by sequencing RT-PCR products derived from each plant. The amino acid residues with 100% homology among the proteins are shown in white character on a black background. The amino acid residues with 50–80% homology have gray background. (C and D) Nodulation phenotype of WT Gifu plants and 30008618 at 14 dai. (E) The position of mutations in lan plants created by the CRISPR-Cas9 genome editing system. Nucleotide alignment of LjLAN is shown. The indel mutations occur near the protospacer adjacent motif (PAM) site (blue letters). The sgRNA target is underlined. (F and G) Nodulation phenotype of WT MG-20 plants, lan #1 and lan #8 at 14 dai. For nodulation analysis of lan #1 and lan #8, T2 generation was used, in which respective homozygous mutation was fixed. NP, nodule primordia. Scale bar: 1 mm. Error bars indicate SD. Tukey’s test was performed by comparing total nodule number. Columns with the same lower-case letter indicate no significant difference. (TIF) [file pgen.1007865.s012.tif]

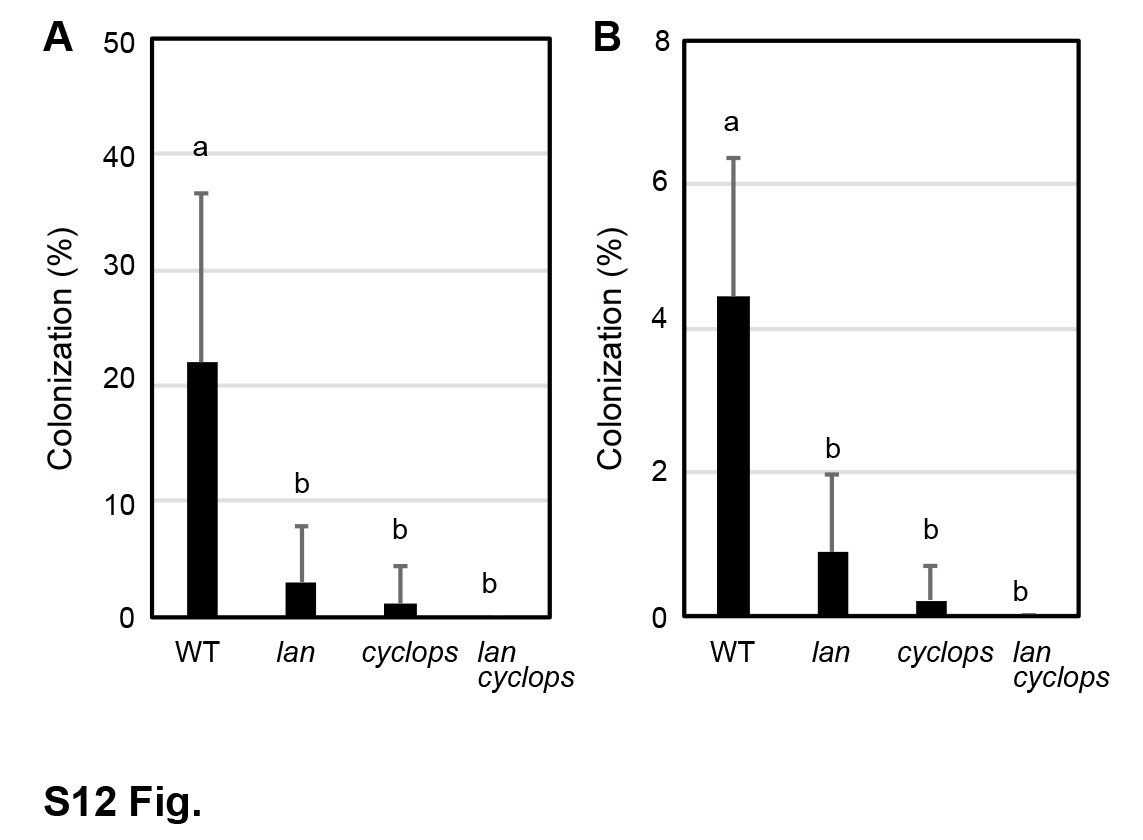

Supplement: S12 Fig — (A and B) R. irregularis colonization ratio of hyphae (A) and arbuscules (B) at 28 dai. (n = 8 plants). Error bars indicate SD. Columns with the same lower-case letter indicate no significant difference (Tukey’s test, P < 0.05). (TIF) [file pgen.1007865.s013.tif]

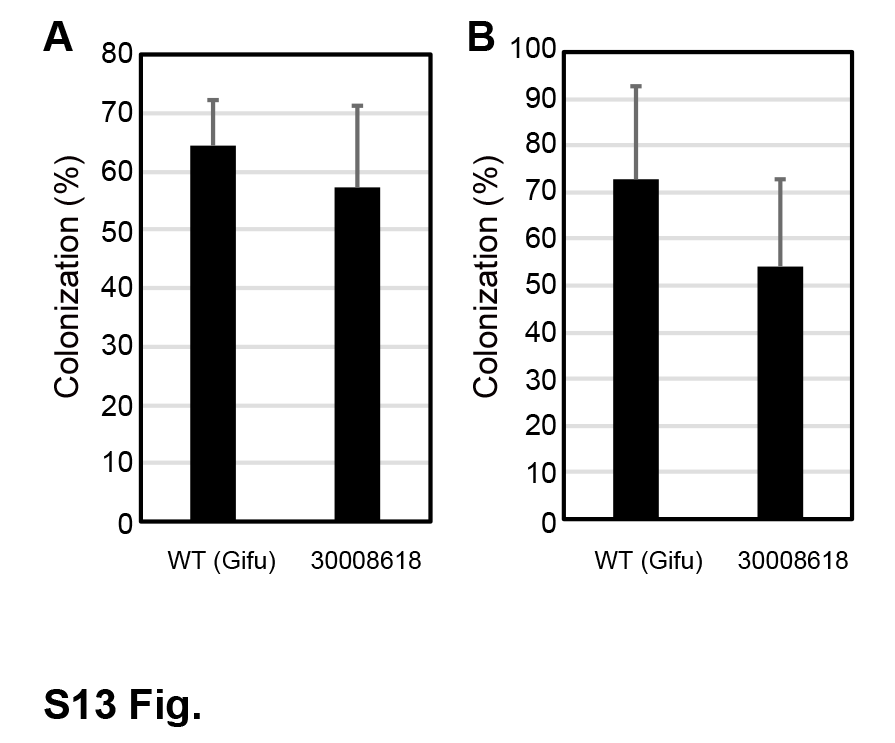

Supplement: S13 Fig — (A and B) R. irregularis colonization ratio of hyphae (A) and arbuscules (B) at 21 dai. (n = 6 plants). Error bars indicate SD. (TIF) [file pgen.1007865.s014.tif]

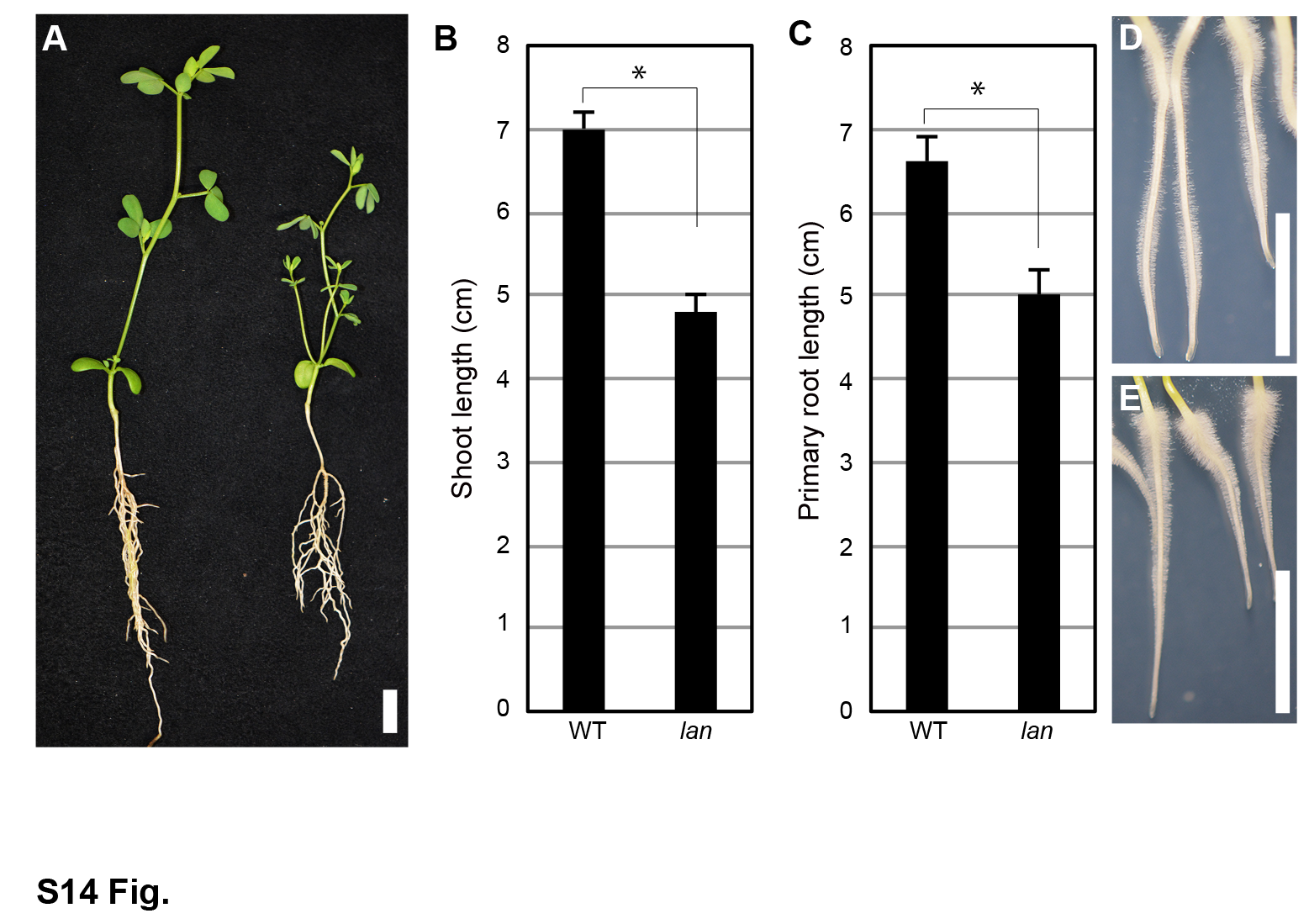

Supplement: S14 Fig — (A) WT MG-20 (left) and lan (right) plants at 14 days after germination (dag). (B and C) shoot (B) and primary root (C) length at 14 dag. Plants were grown in the soil that contained enough nutrients in the absence of rhizobia and AMF. (D and E) Root hairs phenotype of WT MG-20 (D) and lan (E) grown on agar plate at 3 dag. Scale bar: 1 cm. Error bars indicate SD. *P < 0.05 by Student’s t test. (TIF) [file pgen.1007865.s015.tif]

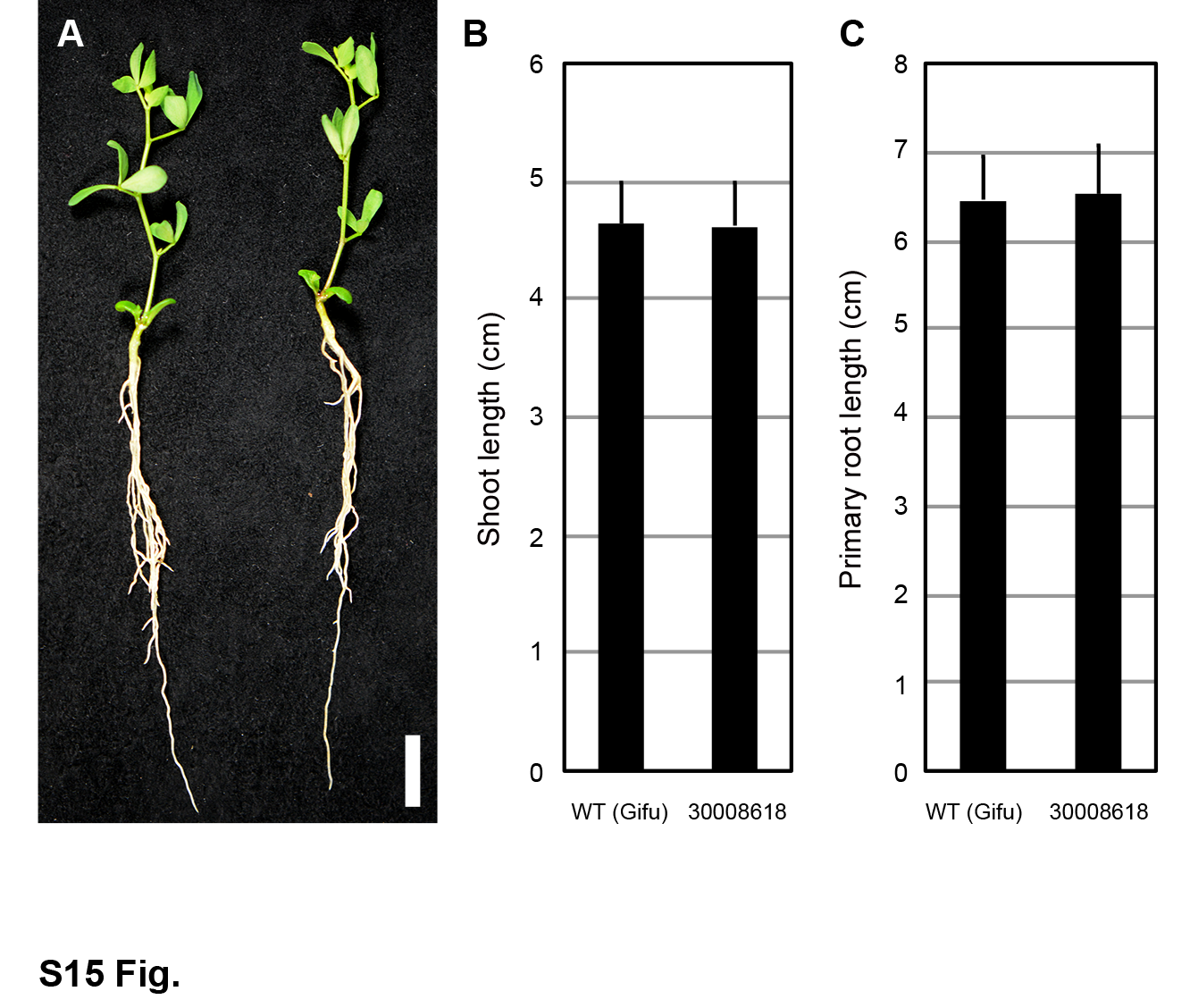

Supplement: S15 Fig — (A) WT Gifu (left) and 30008618 (right) plants at 14 days after germination (dag). (B and C) shoot (B) and primary root (C) length at 14 dag. Plants were grown in the soil that contained enough nutrients in the absence of rhizobia and AMF. Scale bar: 1 cm. Error bars indicate SD. (TIF) [file pgen.1007865.s016.tif]

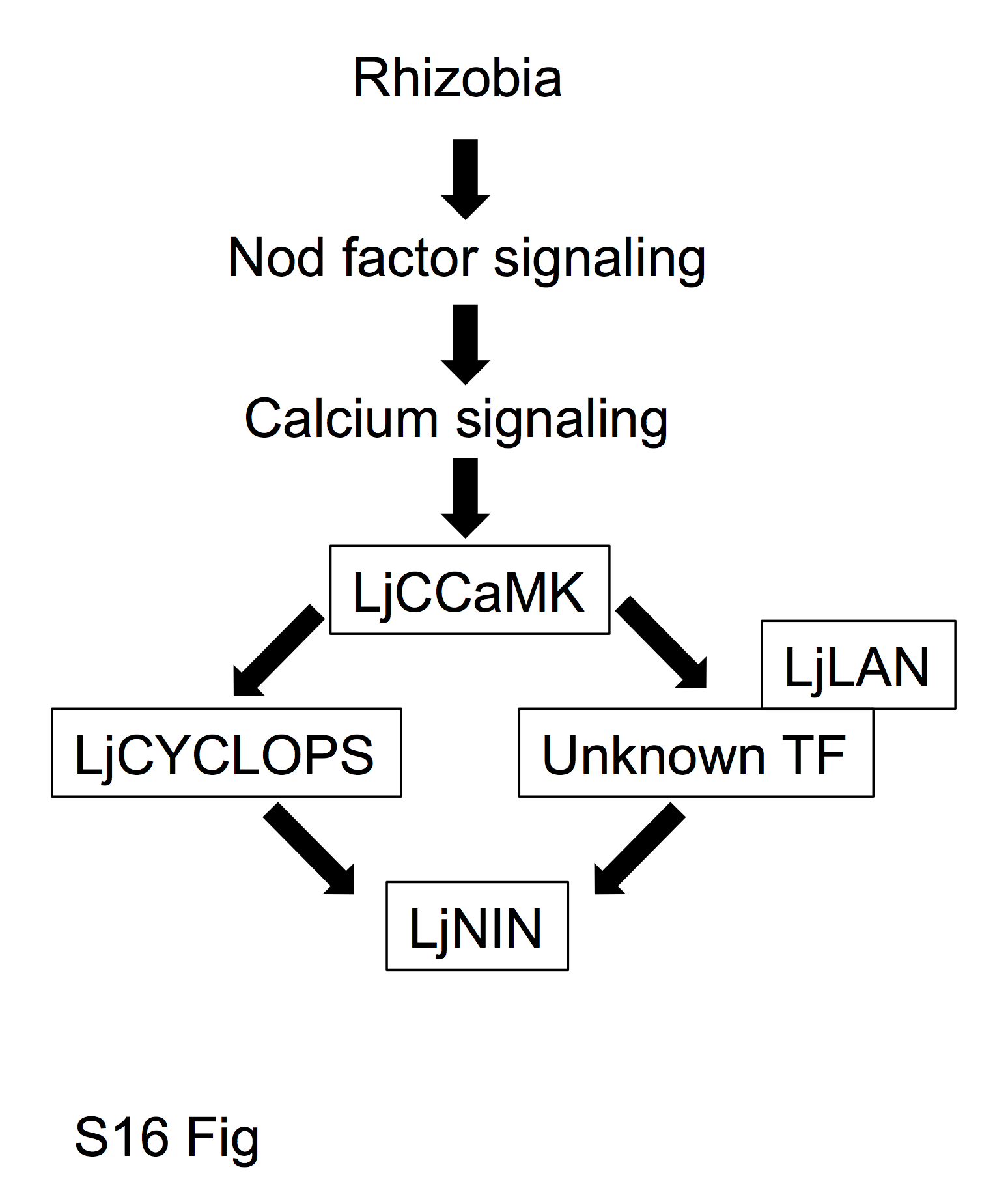

Supplement: S16 Fig — Perception of rhizobia-derived Nod factor by its receptors elicits Nod factor signaling. Consequently, calcium signaling is induced, which is decoded by LjCCaMK. LjCCaMK then activates LjCYCLOPS, which directly induces LjNIN expression. As normal calcium spiking pattern was observed in the lan mutant, the expected position of LjLAN-mediated regulation may be downstream of LjCCaMK. Nodulation phenotype and LjNIN expression in lan cyclops double mutant suggest that LjLAN and LjCYCLOPS act in parallel for the regulation of LjNIN expression. LjLAN, a subunit of Mediator complex, can achieve the regulation by interacting with unidentified transcription factor (TF). (TIF) [file pgen.1007865.s017.tif]
